# Computing the Skyline Query over a *k*^2^-tree compact data structure

**DOI:** 10.1371/journal.pone.0353358

**Published:** 2026-07-17

**Authors:** Rodrigo Torres-Avilés, Gilberto Gutiérrez, Martita Muñoz, Mónica Caniupán

**Affiliations:** 1 Dpto. de Sistemas de Información, Universidad del Bío-Bío, Concepción, Chile; 2 Dpto. Cs. de la Computación y Tec. de Inf., Universidad del Bío-Bío, Chillán, Chile; University of California Riverside, UNITED STATES OF AMERICA

## Abstract

This study aims to explore the feasibility of executing Skyline Queries—used to identify optimal data points based on multiple criteria—within compact data structures. While existing algorithms typically operate on plain or indexed datasets, this work investigates how such queries can be efficiently resolved using compact representations. We focus on implementing two Skyline Query variants, *Constrained Skyline Query* and *Enumerating Skyline*, within the *k*^2^-tree compact data structure. Our approach leverages the *k*^2^-tree’s inherent indexing capabilities to evaluate these queries directly on compressed data, avoiding the need for decompression. The results demonstrate that the *k*^2^-tree structure effectively supports both types of Skyline Queries. Our experimental evaluation shows improved resource utilization compared to non-indexed methods, confirming that compact data structures can process these queries efficiently without data inflation. This article presents a comprehensive study of implementing skyline queries directly over compact data structures—specifically the *k*^2^-tree, supported by extensive experimental evaluation and a complete theoretical analysis of the proposed algorithms.

## 1. Introduction

Information-based decision-making has been highlighted in various areas of human development. As the amount of data and information has grown exponentially, computer decision support/making has become necessary to take the lead in the information era. Moreover, there are different situations in which user decisions are based on a huge amount of multidimensional information and multiple criteria that must be unified to obtain the best options.

The rapid growth of these systems and the increasing size of multidimensional data have led researchers to search for new efficient data processing methods to retrieve useful results [1]. For this reason, the *Skyline Query* has become important in the research of databases to extract interesting objects from multidimensional datasets [2].

**Fig 1:**
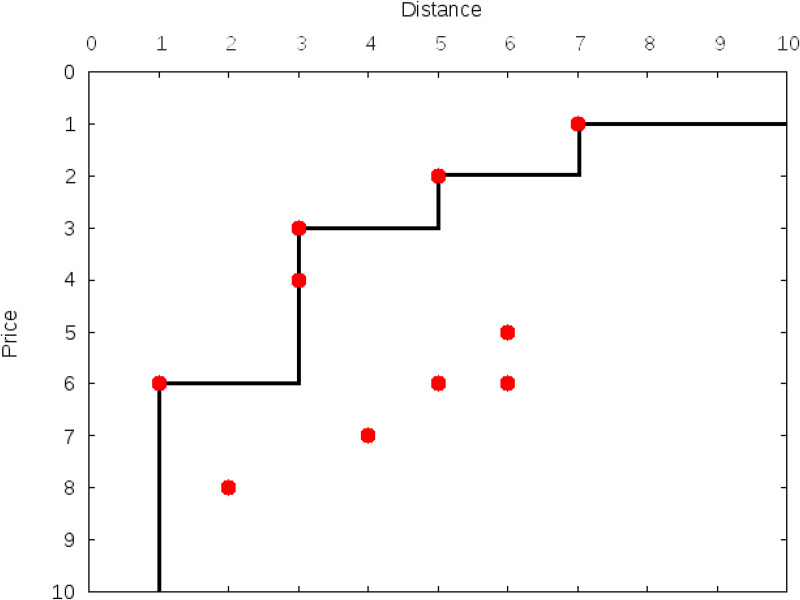
Example of skyline query. Example of points representing houses based on price and distance to a subway station. The points located on the vertices of the black line represent optimal choices, as they are not dominated by any other points. In contrast, the points in the upper-right region of the graph are less favorable options.

Given a set of points, the *Skyline Query* returns all the points that are not *dominated* by other points. Point *a* dominates point *b* if it is as good or better than *b* in all dimensions and it is strictly better than *b* in at least one dimension [3]. User preferences or selection criteria are expressed by comparing two points to identify which is better [4]. As an illustration, consider the challenge of purchasing a house or apartment in a metropolis like Santiago, Chile. Two critical factors to evaluate when selecting a property are *price* and *distance* to a subway station, where lower values for both are generally more desirable. However, these factors often conflict, as properties located closer to subway stations tend to have higher prices. Consequently, the decision involves balancing these competing characteristics based on individual preferences. Fig 1 presents a set of available houses, illustrating this trade-off.

The Skyline Query is a fundamental operator for multi-criteria decision making because it retrieves all Pareto-optimal objects without requiring users to define subjective weighting functions [1]. Its importance was first established by Borzsony et al. [5], who introduced the skyline operator as a way to support intuitive query processing in multi-dimensional spaces. Subsequent work has demonstrated both its theoretical relevance and practical efficiency: Papadias et al. [2] proposed the Branch-and-Bound Skyline algorithm, making skyline computation scalable within database systems, while Lee et al. [6] and Choi et al. [7] introduced advanced indexing and hashing strategies to handle large or high-dimensional datasets efficiently. Additional research has expanded skyline variants, such as reverse skyline queries [8], and explored robustness under incomplete data [9].

The importance of skyline queries is evident across a wide range of domains, such as:

- In decision-making scenarios, they enable users to identify optimal trade-offs among multiple criteria, especially when no single metric is sufficient to determine the best option.
- They also play a key role in applications such as market analysis, business planning, and quantitative economics [10], where evaluating alternatives requires balancing competing objectives.
- Moreover, skyline queries are particularly valuable when users cannot pre-define the relative importance of different attributes, providing a flexible and intuitive framework for multi-criteria evaluation.
- In customer service and recommendation systems, for instance, they can be employed to suggest optimal hotels that are both cost-effective and conveniently located near points of interest [5].
- In bioinformatics, Skyline queries have been applied to DNA sequence search problems, aiding in the identification of optimal matches based on biological criteria [11].
- In the context of location-based services (LBS), they support tasks such as finding the shortest route to a destination or selecting the closest point of interest among many alternatives [12].
- Additionally, a particularly relevant application of this work lies in urban planning for smart cities. Here, decision-makers must identify the most suitable locations for public infrastructure, such as hospitals, schools, or parks, based on multiple criteria including proximity to main roads, air quality, land cost, or accessibility to population centers [13,14].

Thus, these studies demonstrate the pivotal role of skyline queries in multi-objective optimization, recommendation technologies, and data-driven decision-making.

On the other side, the objective of *Compact Data Structures* is to store as much information in the smallest possible space. A key characteristic is that these structures can support queries without uncompressing stored information [15]. Compaction has significant benefits such as maintaining a greater amount of information that would not fit in main memory in its original presentation and improving cache memory performance [15]. This implies that prior methods for non-compact algorithms operating on secondary memory focus primarily on input/output operations, whereas algorithms based on compact data structures emphasize the number of CPU operations.

The *k*^2^-tree compact data structure has been a growing topic of research since its introduction in [16]. It was originally designed to illustrate Web graphs, but it is also useful to represent binary relations. As a *Compact Data Structure*, it stores a greater quantity of information by using less memory. Therefore, a larger amount of data can be processed in main memory avoiding the access to secondary memory.

Even when Skyline Query is centered in multiple criteria (or dimensions), the query remains applicable to two dimensional sets in which *k*^2^-tree is one of the most suitable compact data structures for memory usage. We are interested in process the Skyline query in a two-dimensional set of points stored in a *k*^2^-tree. On static datasets, the Skyline query can be calculated only once and the result can be stored, since this result is invariant. This is especially evident when the query is answered over data over a *k*^2^-tree because it is a static structure (the input set always remains invariant). Thus, it becomes important to get an algorithm that efficiently responds to the *Skyline Query* over a *k*^2^-tree.

There are different variants of the *Skyline Query*. In this article, we focus on two specific and useful variants: *Constrained Skyline Query*, which consists in adding a range in the involved dimensions, and *Enumerating Query*, which consists in counting the number of dominated points of each *Skyline* point.

We introduce two algorithms for the *Constrained Skyline Query* and one algorithm for the *Enumerating Query*, supported by both theoretical and experimental analyses. Building upon [17], this work provides several new contributions: (i) a more detailed and rigorous specification of the proposed algorithms, (ii) an expanded theoretical analysis, and (iii) a comprehensive experimental evaluation, including a comparison with Skyline query processing based on R-tree structures.

Overall, the main contribution of this paper is a substantially extended and more complete study of the first algorithms introduced in [17], with particular emphasis on its implementation using compact data structures and its empirical performance under diverse scenarios. By leveraging the *k*^2^-tree compact data structure, our approach enables the efficient execution of queries directly over compressed spatial data, thereby eliminating the need for decompression and reducing storage overhead. Furthermore, the integration of Constrained Skyline Queries allows for the incorporation of additional spatial or budgetary restrictions, while Enumerating Skyline Queries facilitates the comprehensive identification of all optimal candidates. Overall, this method enhances resource efficiency and query performance, offering a scalable solution for complex decision-making scenarios involving large-scale spatial datasets.

The remainder of the paper is organized as follows. Section 2 reviews the necessary background and related work. Section 3 presents the algorithms for the Constrained Skyline Query, together with their theoretical analysis. Section 4 introduces the algorithm for the Enumerating Query and its corresponding theoretical analysis. Section 5 describes the experimental evaluation, including a comparison with Skyline query processing based on R-tree structures. Finally, Section 6 concludes the paper and outlines directions for future work.

## 2. Background and related work

### 2.1. Skyline Query

The *Skyline Query* is an important operation for many multiple-criteria decision-making applications that has received significant attention in the Database Community [18]. It is a popular and powerful paradigm that allows extracting points of interest from a multidimensional dataset [19].

**Definition 1.** *Let q and p be two d-dimensional points defined as* $q=(q_{1},...,q_{d})$ *and* $p=(p_{1},...,p_{d})$*, and without any loss of generality and assuming that the lowest values in each attribute are the best. The dominance relation* q≻p *is defined as:*


$$\begin{array}{c} q≻p\Leftrightarrow \left(\overset{d}{\underset{i=1}{⋀}}q_{i}\leq p_{i}\right)\wedge \left(\overset{d}{\underset{i=1}{⋁}}q_{i}<p_{i}\right) \end{array}$$

**Definition 2.** *Let D be a set of d-dimensional points. The Skyline* S⊆D *is defined as:*


$$\begin{array}{c} S=\{p\in D:∄q\in D:q≻p\} \end{array}$$

Although the *Skyline Query* has been studied since the 1960s, it has been rediscovered in the context of databases recently. Borzsony et al. [5] propose an extension in SQL (Structured Query Language), to process the query and the first algorithms to solve this problem in databases. A series of algorithms have since emerged that respond to the *Skyline Query* in secondary memory. These algorithms can be classified in two main groups [19]:

- **Sequential or generic algorithms:** which do not require the input set to be indexed in any data structure. One algorithm is BNL [5], which searches the whole set, stores the points it considers that belong to the *Skyline* in a buffer, and updates the buffer as it searches through the points in secondary memory. Variants of this algorithm are SFS [20] and SaLSa [19,21], which preorder the set to reduce dominance comparisons.
- **Algorithms based on indices or other structures:** which require the input set to be indexed in some data structure to take advantage of its properties and reduce the number of dominance comparisons in advance. They use *B*-trees [22], Bitmaps [22], and *R*-trees [23].

In the second class of algorithms, the *Branch and Bound Skyline algorithm* (BBS) [2,24] is the most efficient strategy encountered in the literature. It presents a series of properties allowing the reduction of the search space, and is also suitable for working in interactive environments [1,23]. It runs through the nodes of an *R*-tree and avoids expanding nodes that do not contain *Skyline* points.

The BBS algorithm uses a minimal Heap *H* of MinDists. A minimal Heap (or MinHeap for short) is a binary tree in which every internal level is kept full and every key of each node is less or equal to the keys of its children. The key stored for the BBS algorithm is the MinDist, which is simply the minimal distance between the node and the origin. In the case of *R*-trees, the nodes are MBR (Minimal Bounding Rectangle), and the MinDist is calculated by the minimal distance of the rectangle to the origin, whose corresponding point is not necessarily a point of the *R*-tree and has to be further expanded. MinHeaps have an efficient (logarithmic in terms of its length) insertion and delete query times.

The following steps describe how the algorithm works:

1. The root of the structure (as *R*-tree) is expanded and its children are inserted in *H*.
2. The *e* entry on the top is extracted from *H*.
3. If *e* is dominated by some element of *S*, it is discarded.
4. If *e* is an internal node and is not discarded, it is expanded (consider $e_{i}$ as the i-th child of *e*). The dominance check of each node $e_{i}$ is performed in relation to *S*. If it is dominated, $e_{i}$ is discarded. If not, $e_{i}$ enters *H*.
5. If *e* is a point not dominated by any element of *S*, it is entered in *S*.
6. The algorithm ends when *H* is empty.

### 2.2. Skyline Query variants

A large number of variants of this query have emerged in the literature. Each variation is applicable and can solve different aspects of a problem. The BBS algorithm, in addition to its efficiency, can be adapted to easily respond to some of these variants without generating an excessive additional cost [2]. One variants is the *Constrained Skyline Query*.

**Definition 3.** *Let D be a set of d-dimensional points and* r⊆D *a hyper-rectangle within D. The Constrained Skyline Query asks for the set* S⊆r *defined as:* S={p∈r:∄q∈r:q≻p}*.*

This variant delivers the *Skyline* points and dominates the points within a space defined by a series of restrictions, usually expressed as a hyper-rectangle [2]. For example, this query is useful when one of the dimensions is price and the query needs a minimum and/or maximum budget and/or when one of the dimensions is distance and it is possible to specify a distance range in the *Skyline Query*. This is how a new *Skyline* can be obtained based on the new restrictions and existing preferences. The BBS algorithm can be adapted to answer this variant by including the intersection between the *e* node and the range query as an additional condition in step 5 [2].

Another variant is *Enumerating Skyline Query* (or simply *Enumerating Query*).

**Definition 4.** *Let D be a set of d-dimensional points and* S⊆D *its Skyline points. The Enumerating Skyline Query asks for the set of pairs* SS⊆S×ℕ *defined as:*


$$\begin{array}{c} SS=\{s,d\in D\times \mathbb{N}:s\in S\text{ and }d=|\{p\in D:s≻p\}|\} \end{array}$$

This query returns the number of points dominated by *s* (called *num*(*s*)) for each point of the *Skyline* s∈S [2]. This information may be relevant in applications where the *goodness* of a point on the *Skyline* is determined by the number of dominated points [2]. To solve this variant, the conventional *Skyline* is calculated using BBS and then the number of points contained within the *s* dominance region is counted for each *s* point belonging to the *Skyline*.

### 2.3. *k*^2^-tree

The *k*^2^-tree compact data structure compactly stores binary sparse matrices [15]. It was originally shown in [16] to store Web graphs in an adjacency matrix. This structure is designed to compress large areas with zeros (no relation) [16] and improve clustered data compaction.

For binary relations, such as a Cartesian plane ℕ×ℕ, the origin of the coordinate axis is located in the upper left corner of the matrix, while columns represent the *x*-axis and rows the *y*-axis starting at zero [15]. A point (*i*,*j*) in the Cartesian plane is then represented as a 1 in the (*i*,*j*) position in the adjacency matrix. If the points are on a different scale (or they are in ℝ×ℝ), they can be re-scaled to fit in a n×n adjacency matrix, taking care of the level of precision needed.

**Fig 2:**
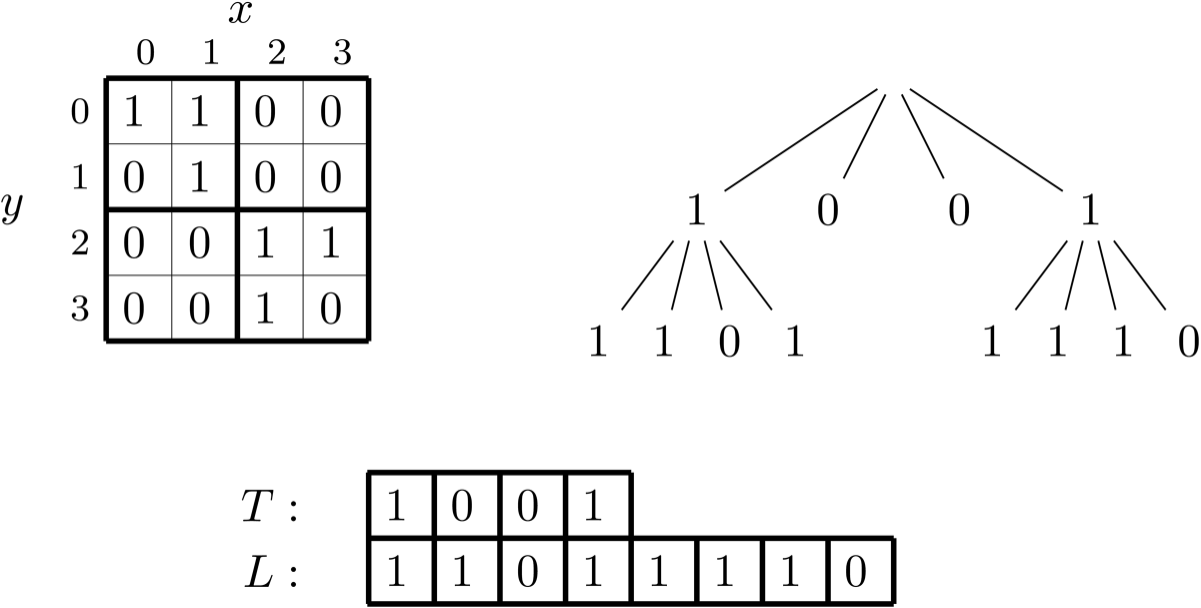
Example of a *k*^2^-tree. In the upper left, the matrix representation of the points. In the upper right, its *k*^2^-tree representation. At the bottom, the *k*^2^-tree as bitmaps *T* and *L.*

Let *M* be a matrix with dimensions n×n where *n* is a power of *k*. The matrix is subdivided into *k*^2^ submatrices of $\frac{n}{k}\times \frac{n}{k}$. Submatrices are counted from left to right and from top to bottom. Each *k*^2^ submatrix is represented by a bit whose value is 1 if it contains at least one point or 0 if it is empty. Submatrices with a value of 1 are recursively subdivided into *k*^2^ submatrices. Subdivision ends when an empty submatrix is found or when the individual cells are reached. Fig 2 shows an example of using a *k*^2^-tree where *k* = 2 and *n* = 4.

Any operation over a *k*^2^-tree requires two basic operations over the bitmaps *T* and *L*, which are typical of compact data structures. Let *B* be a bitmap of size *l* and the operation $rank_{b}(B,i)$ counts the number of bits of *B* whose value is *b* (zero or one) to position *i* with 1≤i≤l. In addition, the operation $select_{b}(B,i)$ calculates the position of *B* to where there are *i* bits with value *b* [15,16].

The operation *rank* can be calculated in time *O*(1) and *selected* in time O(loglogn) using storage *n* + *o*(*n*) [16]. Storage is used for both the bitmap and the additional structure that reduces the response time of both queries. Using these operations on the bitmaps, it is possible to efficiently pass through the *k*^2^-tree to obtain the children of a submatrix [16]. It is also possible to efficiently count points stored in a *k*^2^-tree in a given range *r* with an expected time of $O(log_{k}r\cdot log_{k}n)$ [25].

To our knowledge, there is no implementation of the Skyline Query on compact data structures. It is therefore needed to obtain all the points (uncompress the data) and apply a known algorithm in these non-indexed points.

We show that computing the *Skyline Query* over a *k*^2^-tree compact data structure can be done by significantly decreasing the number of dominance comparisons, thus reducing data processing and the amount of resources used in the Skyline Query. Initially, one can easily compare dominance at the bit level between sibling nodes and discard those dominated by any sibling. More than $(k-1{)}^{2}$ submatrices can be discarded.

## 3. Algorithms for constrained Skyline Query

### 3.1. Algorithm *BBSkConstrained*

Algorithm *BBSkConstrained* responds to the *Constrained Skyline Query* variant on indexed data in a *k*^2^-tree based on the BBS strategy. This is it uses a heap to store the submatrices data that contain points of the solution to revise later according to their distance to the origin. Information about the stored submatrices are the submatrix index *i* in the bitmap *T*, vertex (*x*, *y*) nearest to the submatrix origin (called *MINDIST(i)*), and submatrix size. It is important to point out that the MinDist of a submatrix (which is the distance between the upper left vertex of the square) is not necessarily the real minimal distance of the set of points inside the submatrix because the upper left vertex of the square could be empty. Each submatrix extracted from the heap is verified so that it is not dominated by any point already ahead in the *Skyline*. For each expanded submatrix, its *k*^2^ children are revised in the same order in which they are located in the *k*^2^-tree. The search uses four discard conditions that determine whether the submatrix enters the heap (or the solution set if it is a leaf) or is discarded. The submatrix or leaf is discarded if one of the following occurs: (i) It is dominated by a *Skyline* point that has already been found. (ii) It is empty. (iii) It is dominated by some non-empty sibling. (iv) It is outside the range query.

The third discard condition can only be applied if the parent of the submatrix to be revised is completely contained within the range query. On the contrary, the sibling discarding the current submatrix could not have a *Skyline* point within the range query (*i.e.,* the point that dominates the current submatrix could not be inside the range).

**Algorithm 1. BBSkConstrained (*k*, *r*)**.

**Input:** *K* The *k*^2^-tree structure

**Input:** *r* The range query

**Output:** The Constrained Skyline Query answer

1: Insert *root* in *heap*

2: S=∅

3: **while** heap≠∅ **do**

4:  η= *heap* top

5:  **if** η is not dominated by *S* **then**

6:   **for** each child $\eta_{i}$ in η **do**

7:    **if** $\eta_{i}\text{≠}\emptyset \text{ and }\eta_{i}$ is not dominated by *S* **then**

8:     **if** η⊆r **then**

9:      **if** $\eta_{i}$ is not dominated by its non-empty sibling **then**

10:       **if** $\eta_{i}$ is not a leaf **then**

11:        $heap=heap\cup \eta_{i}$

12:       **else**

13:        $S=S\cup \eta_{i}$

14:     **else**

15:      **if** $\eta_{i}\cap \text{r}\;\text{≠}\;\emptyset$ **then**

16:       $heap=heap\cup \eta_{i}$

17: **return** *S*

Algorithm 1 illustrates *BBSkConstrained*. Line 7 corresponds to the application of the first two discard conditions. Line 8 applies the precondition required for the third condition applied in the next line. This precondition checks that node $\eta_{i}$ is inside region *r*. Line 15 shows that the application of the fourth discard condition can be observed for those submatrices whose parents are not inside the range *r*.

**Fig 3:**
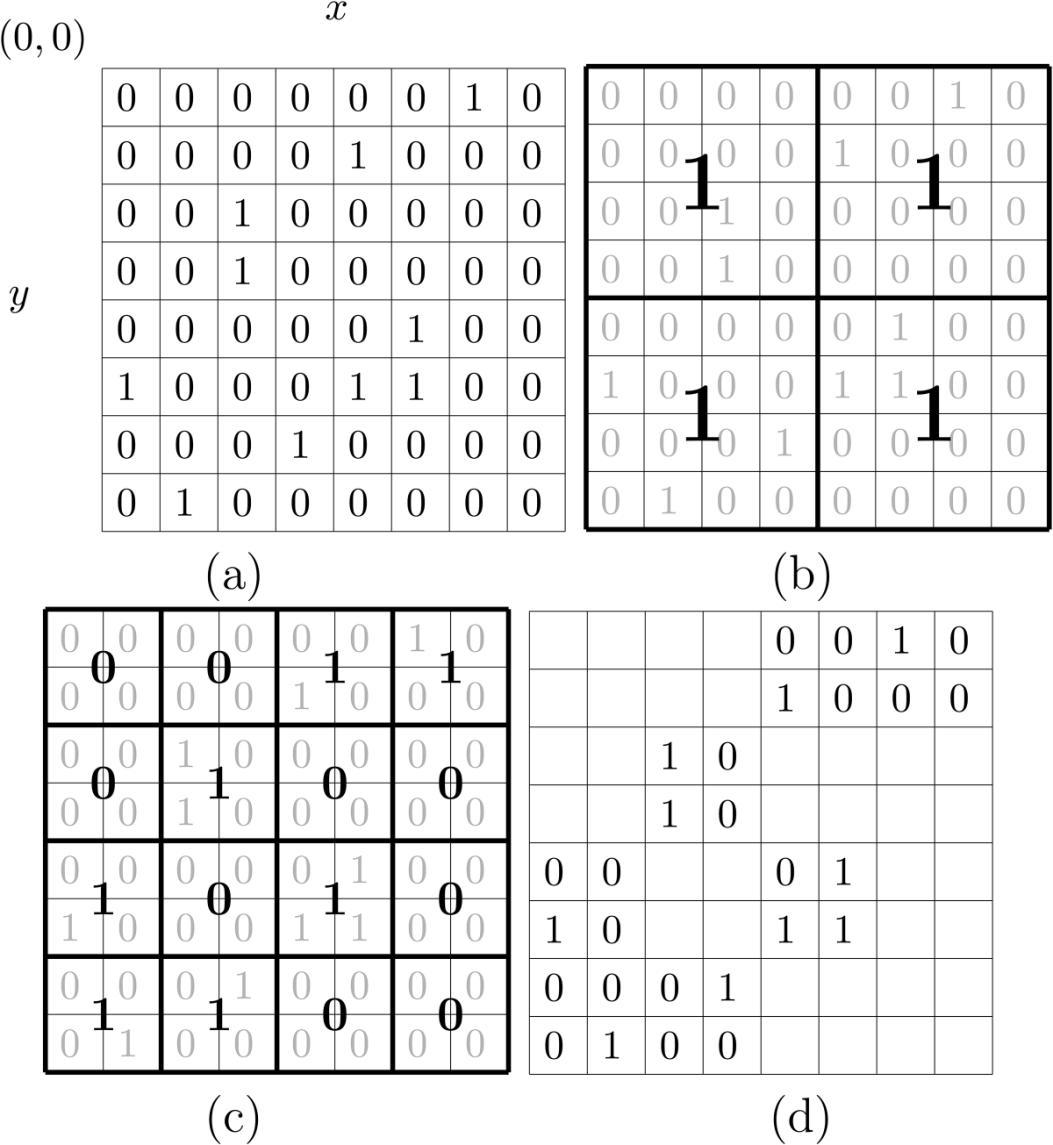
Example to run the algorithms. (a) Points *P* represented in matrix form. (b) Level 1 of the *k*^2^-tree of *P*. (c) Level 2 of the *k*^2^-tree of *P*. (d) Leaf level of the *k*^2^-tree of *P*.

**Fig 4:**
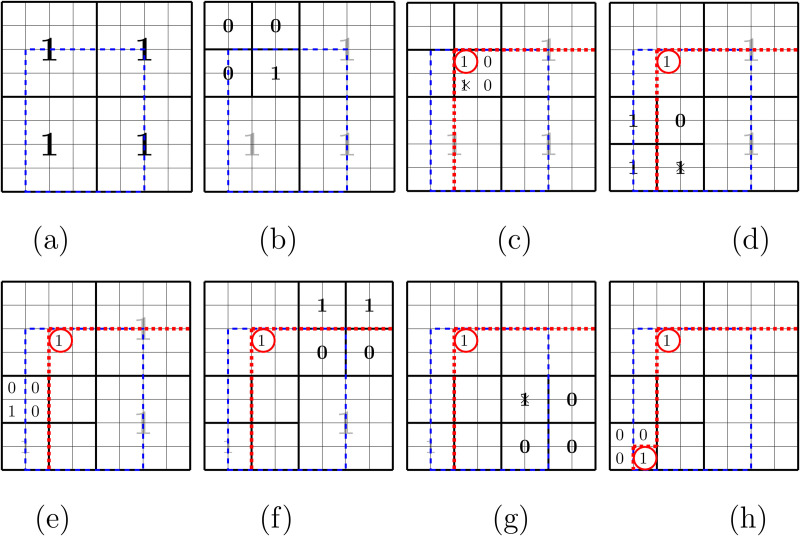
*BBSkConstrained* running example. (a) Checking the root matrix. (b) Checking the first non-empty child. (c) Finding the first point. (d) Pruning a dominated submatrix. (e) Discarding empty cells. (f) Discarding empty submatrices. (g) Pruning a dominated submatrix. (h) Finding a second point.

**Example 1.** *As an illustration,* Fig 3 *shows a set of points represented in a matrix* (Fig 3(a)). Fig 3(b) *is the level 1 of the k*^*2*^*-tree representation overlaying the points for k = 2.* Fig 3(c) *shows level 2 of the k*^*2*^*-tree representation overlaying the points.* Fig 3(d) *shows the leaf level of the k*^*2*^*-tree representation.* Fig 4 *shows the execution of Algorithm 1 considering the range query* r=[1,5]×[2,7]*. In the graphics, the range query is depicted as a dashed square, the Skyline points are circled, the area dominated by the Skyline set is represented as a dotted polygon, and any point discarded by domination is marked with a* × *symbol. Additionally, every node processed in the current while loop iteration is highlighted. In the following paragraphs, we describe each iteration of the while loop in Algorithm 1.*

*At the beginning of Algorithm 1, the root node is inserted into the heap. During the first iteration of the while loop (Line 3), the first child at level 1 (upper-left node) is processed in the for loop (Line 6) (see* Fig 4(a)*). The condition in Line 7 does not apply yet because S is empty. Since this node is not completely contained within the range (Line 8) but intersects it, it is added to the heap. The same process applies to all other nodes at level 1 (see* Fig 3(b)*).*

*In the next iteration of the while loop, the first element in the heap is the upper-left node at level 1 (see* Fig 4(b)*). This node has only one non-empty child,* $\eta_{4}$*, located at the bottom right, which is entirely within the range. The conditions in Lines 7 and 9 remain inapplicable because S is still empty, and* $\eta_{4}$ *has no siblings. Consequently,* $\eta_{4}$ *is added to the heap. At this point, the heap contains four elements: the upper-right, bottom-right, and bottom-left nodes from level 1, along with the recently added node from level 2. The latter has the smallest MINDIST() value and is therefore popped from the heap. Its first leaf, at position (2,2), is not discarded by any of the conditions in Lines 7, 8, or 9, as S is still empty. Since it has no siblings and lies within the range, it is added to the Skyline set in Line 13 as its first point (see* Fig 4(c)*). The second leaf of this node is discarded because it is dominated by the first Skyline point.*

*Now, the upper-right and bottom-left nodes at level 1 have the same MINDIST() value. First, consider the bottom-left node. It has three non-empty children, of which two intersect with the range and are added to the heap and the third child is discarded in Line 7 because it is dominated by the Skyline point (see* Fig 4(d)*).*

*In the next two iterations, all child nodes are discarded because they lie outside the range (see* Fig 4(e) *and* Fig 4(f)*).*

*The next node at the top of the heap is the bottom-right node at level 1, whose only non-empty child is dominated by S (see* Fig 4(g)*). Finally, the last node in the heap has one non-empty child within the range that is not dominated by S. This child is added to S (see* Fig 4(h)*). Once the heap becomes empty, the algorithm terminates, yielding the result S = (2,2), (1,7).*

### 3.2 Algorithm *BBSkConstrained*: Theoretical analysis

The following lemma allows us to show that the Algorithm 1 is correct.

**Lemma 1.** *Any point within a discarded submatrix cannot be a Skyline point for the range r.*

*Proof. A point* s∈S *is said to dominate an entire submatrix* η *if every possible point* p∈η *is dominated by s, i.e.,* ∀p∈η:s≻p*. It is important to note that the exact points within the submatrix are unknown while traversing the tree. Therefore,* s≻η *holds if the nearest possible point to the origin within* η *(whether empty or not) is dominated by s; a condition that can be efficiently verified.*

*Similarly,* $\eta_{1}≻\eta_{2}$ *if the farthest point to the origin in* $\eta_{1}$ *dominates the nearest point in* $\eta_{2}$*. This condition ensures that* $\eta_{1}$ *must lie in the upper-left direction relative to* $\eta_{2}$*. For instance, if* $\eta_{1}$ *is only above but not to the left of* $\eta_{2}$*, then a point* $p_{2}\in \eta_{2}$ *could exist such that* $\forall p\in \eta_{1}:p_{2}.x>p.x$*. In this case, p*_*2*_ *remains a potential Skyline point.*

*Finally, the second and fourth discard conditions follow directly from these observations.* □

We now state the following theorem.

**Theorem 1.** *Algorithm 1 is correct.*

*Proof. Let us to prove that every point added to S during the execution of the algorithm is a final skyline point. A point* $\eta_{i}$ *is added to the Skyline in Line 13. Suppose there exists a point p that dominates* $\eta_{i}$ *and satisfies* $p.x<\eta_{i}.x$ *and* $p.y<\eta_{i}.y$*. In this case,* $\eta_{i}$ *must belong to a submatrix discarded by p or a submatrix containing p; thus,* $\eta_{i}$ *would never be added in Line 13. If* $p.x\leq \eta_{i}.x$ *and* $p.y<\eta_{i}.y$*, or* $p.x<\eta_{i}.x$ *and* $p.y\leq \eta_{i}.y$*, the point p must first be added to the Skyline set S because the order in which points are visited is determined by their distance from the origin. Consequently,* $\eta_{i}$ *would never be added in Line 13, as it would be discarded in Line 7.*

*Finally, every discarded point is not a Skyline point, as demonstrated in Lemma 1.* □

Let the size of the matrix represented in the *k*^2^-tree be (n×n), the number of points be *m* = |*P*|, the number of children for each node be *k*^2^, and the set *S* be the Skyline set. To estimate the theoretical resources used by Algorithm *BBSkConstrained*, we first need to determine the number of nodes entering the heap for specific actions in order to calculate the total number of nodes to be processed. To simplify the analysis, we consider the entire matrix as the range query. Therefore, the height of the tree is $h=\log_{k}n$. However, a tighter upper bound can be derived if the range is smaller.

Since the structure is a tree, we only need to consider the following results: the lower height *H* (the height of the lowest node where the range is fully contained) and add the time to reach this range, expressed as O(h−H).

The following lemma allows us to quantify the children inserted in the heap for each internal node.

**Lemma 2.** *For each expanded node, at most* 2k−1 *of its k*^*2*^ *children are inserted in the heap.*

*Proof. This is a geometric problem. Consider a grid of* k×k *cells, where each cell represents a child of one node. The maximum number of cells (nodes) that are not dominated by any other corresponds to the cells along the upper-left perimeter, as any other cell has a greater value along at least one of the axes. This number is exactly* 2k−1 *cells, assuming that each node is non-empty.*

An important question arises: how many nodes are expanded? At first glance, this question might seem impossible to answer directly. However, we can confidently state that if a node contains a Skyline point, it must be expanded until a leaf is reached, as such a node cannot be discarded.□

The following lemma allows us to quantify of amount of added nodes to the heap by level.

**Lemma 3.** *When descending through a node i containing at least one Skyline point, and continuing until that point is reached, the heap grows by at most* $2(k-1)h_{i}+1$ *nodes, where* $h_{i}$ *is the height of the node.*

*Proof. When descending through the node to the first level, at most* 2k−1 *nodes are added, as stated in Lemma 2. For each subsequent lower level, the heap pops one node and pushes at most* 2k−1 *nodes. This results in a net addition of* 2k−2 *new nodes per level.*

*Therefore, the total number of nodes added to the heap is at most:*


$$\begin{array}{c} 2k-1+(2k-2)(h_{i}-1)=2(k-1)h_{i}+1. \end{array}$$

As a consequence of Lemma 3, we obtain the following theorem.□

**Theorem 2.** *The maximum number of visited nodes is given by*


$$\begin{array}{c} \sum_{s_{i}\in S}(2(k-1)h_{s_{i}}+1), \end{array}$$

*where* $h_{s_{i}}$ *is the height of the highest node containing* $s_{i}$ *but not containing any* $s_{j}$ *with j < i.*

On average, other expanded nodes do not descend much further because they are rapidly discarded by the previously computed Skyline points. This effect is particularly pronounced since the first Skyline point, *s*_1_, is the closest to the origin.

We can state that $h_{s_{1}}=h$, but the height of every other Skyline point depends on how clustered the points are in the space. If we suppose that, in the worst case scenario, $h_{s_{i}}=h-(i-1)$, we obtain:


$$\begin{array}{c} \sum_{s_{i}\in S}(2(k-1)h_{s_{i}}+1)=O(|S|\cdot k\cdot (\log_{k}n-|S|)) \end{array}$$

When points are more densely clustered, the average number of children expanded per node decreases because many of the 2k−1 potential children at higher levels are empty. Based on this observation, the best case scenario is: $O(\log_{k}n+|S|)$.

In a typical case for a *k*^2^-tree (commonly used for clustered data), most upper levels have empty nodes, with a higher density of points concentrated in the lower levels.

If we suppose that, for example, $h_{s_{i}}=\frac{h}{2^{i-1}}$, we obtain:


$$\begin{array}{c} \sum_{s_{i}\in S}(2(k-1)h_{s_{i}}+1)=O(k\cdot \log_{k}n+|S|) \end{array}$$

When analyzing resource consumption over time, we must consider the cost of applying the four discard conditions. All conditions, except the first, have a computational cost of *O*(1). The first discard condition requires comparing the current node with all previously identified Skyline points, resulting in a worst-case cost of *O*(|*S*|). Additionally, each node must be inserted into and removed from the heap, which incurs a logarithmic cost relative to the heap size.

Therefore, the asymptotic time complexity of this algorithm ranges from


$$\begin{array}{c} O(|S|\cdot (\log_{k}n+|S|)\cdot \log_{2}(\log_{k}n+|S|)) \end{array}$$

to


$$\begin{array}{c} O(|S{|}^{2}\cdot k\cdot (\log_{k}n-|S|)\cdot \log_{2}(|S|\cdot k\cdot (\log_{k}n-|S|))), \end{array}$$

with an expected case of


$$\begin{array}{c} O(|S|\cdot (k\cdot \log_{k}n+|S|)\cdot \log_{2}(k\cdot \log_{k}n+|S|)). \end{array}$$

For spatial storage costs, the heap is the most significant contributing structure. Thus, the spatial complexity ranges from $O(\log_{k}n+|S|)$ to $O(|S|\cdot k\cdot (\log_{k}n-|S|))$, with an expected case of


$$\begin{array}{c} O(k\cdot \log_{k}n+|S|). \end{array}$$

### 3.3. Algorithm *SkylineX*

We present a second alternative to the *Constrained Skyline Query* variant on indexed data in a *k*^2^-tree. It works in a way that is very similar to *BBSkConstrained*, except for two differences. The first is the order exhibited by the heap. Submatrices are ordered in relation to the smallest value in the *x*-coordinate of the node. If two submatrices have the same *x*-value, they are ordered in relation to the smallest *y*-coordinate.

**Fig 5:**
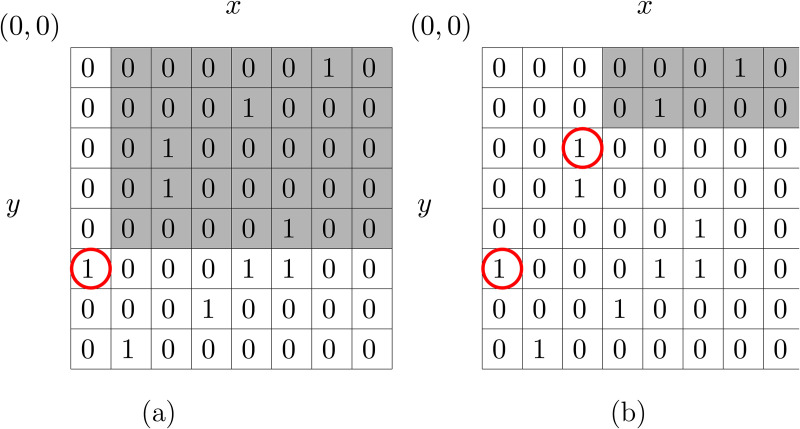
Skyline X. Update of *R* (highlighted) while more *Skyline* points are found. (a) *R* after the first Skyline point. (b) *R* after the second Skyline point.

The second difference is the union of the first and fourth discard conditions. Initially, we define rectangle *R* as equivalent to the range query *r*. Each time a *Skyline* point is located, *R* is updated by placing its lower left corner in the next upper right cell of the *Skyline* point that was found. The next *Skyline* point can only be found within *R*. Fig 5 provides an example of the *R* update while more *Skyline* points are found.

**Algorithm 2. SkylineX(*k*, *r*)**.

**Input:** *K* The *k*^2^-tree structure

**Input:** *r* The range query

**Output:** The Constrained Skyline Query answer

1: Insert *root* in *heap*

2: S=∅

3: *R* = *r*

4: **while** heap≠∅ **do**

5:  η= *heap* top

6:  **if** η∩R≠∅ **then**

7:   **if** η is a leaf **then**

8:    S=S∪n

9:    Readjust *R*

10:   **else**

11:    **for** each child $\eta_{i}$ in *n* **do**

12:     **if** $\eta_{i}\text{≠}\emptyset \text{ and }\eta_{i}\cap R\text{≠}\emptyset$ **then**

13:      **if** $\eta \subseteq r\text{ and }\eta_{i}$ is not dominated by its non-empty sibling **then**

14:        $heap=heap\cup \eta_{i}$

15: **return** *S*

Algorithm 2 expresses the pseudocode of *SkylineX*. Before subdividing node η, it is initially checked to see if it intersects *R*, as seen in Line 6 because this range could have changed while node η was in the heap. Line 12 is the application of the second discard condition over child $\eta_{i}$, which is checked again to see if $\eta_{i}$ intersects *R*. Line 13 is the application of the third discard condition after verifying the required precondition.

**Fig 6:**
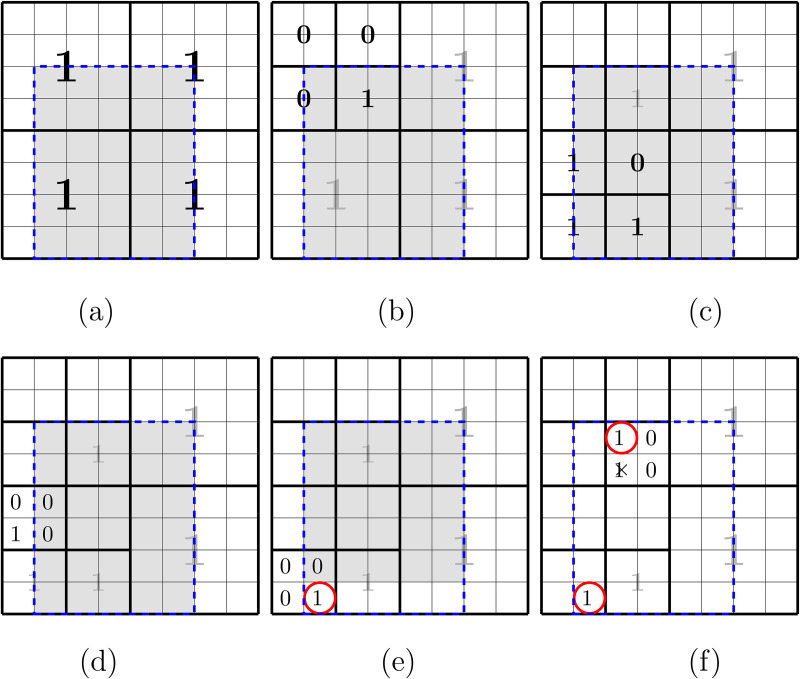
*SkylineX* running example. (a) Checking the root matrix. (b) Checking the first non-empty child. (c) Checking the second non-empty child. (d) Discarding empty cells. (e) Finding the first point. (f) Finding a second point.

To explain the algorithm, we use the same input setting as in Example 1, and provide explanations for each iteration of the outer cycle (*while*) in Algorithm 2. Fig 6 illustrates the iterations. Again, the range query is depicted as a dashed square, *R* is the highlighted square, the points in the Skyline are circled, and a discard by domination is indicated by a × symbol. Finally, all nodes involved in the current *while* cycle are highlighted.

**Example 2.** *At the beginning of Algorithm 2, the root is inserted into the heap, and its children are evaluated because it intersects with R and is not a leaf (see* Fig 6(a)*). Each of its children is non-empty and intersects with R (Line 12). However, since the root is not completely contained within the original range r, we cannot apply sibling-based discarding (Line 13). Therefore, all of its children are added to the heap.*

*In the next iteration of the while loop, the first element of the heap is again the upper-left node in level 1 (see* Fig 6(b)*). Its only non-empty child intersects with R (Line 12) and is fully contained within the original range r. It is not discarded by its siblings (Line 13) and is therefore added to the heap.*

*Then, we observe the first distinction from Algorithm 1. The top of the heap is now the bottom-left node in level 1, since the selection is determined by the distance along the x-axis (see* Fig 6(c)*). All its non-empty children intersect R, but since their parent is not fully contained within r, they are all added to the heap. However, the uppermost node has no children within R and therefore they are discarded (see* Fig 6(d)*).*

*The next leftmost element in the heap is the bottom-leftmost node in level 2. Its only non-empty child is not discarded by either Line 12 or Line 13. In the next iteration, this child is a leaf and becomes the first Skyline point, prompting an update of R (see* Fig 6(e)*).*

*Once again, the heap pops the leftmost node in the heap. Since there are two candidates, the uppermost node is selected (see* Fig 6(f)*). This node has two non-empty children, both of which are inside R and r, and are therefore added to the heap. In the subsequent cycle, the uppermost child of this node is popped first. As it is a leaf, it becomes the second Skyline point. With this update, R becomes empty, and the next element in the heap is discarded due to Line 6.*

*From this point onward, with R now empty, the remaining three nodes in the heap are discarded as they are popped, as dictated by Line 6, and the algorithm finished.*

### 3.4. Algorithm *SkylineX*: Theoretical analysis

Since the first discard condition is replaced by the intersection with *R*, the cost of evaluating a node is always constant. Therefore, the computational cost is directly associated with the number of nodes to be processed. To reach a Skyline point, we must descend to a leaf; thus, we can use the result in Theorem 2 where the number of expanded nodes is given by:


$$\begin{array}{c} \sum_{s_{i}\in S}(2(k-1)h_{s_{i}}+1), \end{array}$$

where $h_{s_{i}}$ represents the height of the highest node containing $s_{i}$ but not containing $s_{j}$ for *j* < *i*.

We can affirm that $h_{s_{1}}=h$, but because the first Skyline point is the leftmost point rather than the nearest to the origin, it is possible that more $h_{s_{i}}$ values are closer to the top of the tree. Based on these observations, the best- and worst-case scenarios with respect to the amount of expanded nodes remain the same as in the previous algorithm: from $O(\log_{k}n+|S|)$ to $O(|S|\cdot k\cdot (\log_{k}n-|S|))$.

However, the expected case differs from the previous one. As mentioned earlier, it is likely that a fraction of the Skyline points will have higher $h_{s_{i}}$ values compared to the previous algorithm, approaching the height of the tree. Consequently, the expected asymptotic behavior aligns with the worst-case scenario: $O(|S|\cdot k\cdot (\log_{k}n-|S|))$.

Each node must enter and leave the heap, leading to a temporal cost that ranges from


$$\begin{array}{c} O((\log_{k}n+|S|)\cdot \log_{2}(\log_{k}n+|S|)) \end{array}$$

to


$$\begin{array}{c} O(|S|\cdot k\cdot (\log_{k}n-|S|)\cdot \log_{2}(|S|\cdot k\cdot (\log_{k}n-|S|))). \end{array}$$

For spatial cost analysis, the heap is the dominant factor. Thus, the spatial cost exhibits the same asymptotic behavior as the temporal cost. The best-case scenario is $O(\log_{k}n+|S|)$, while the expected and worst cases are $O(|S|\cdot k\cdot (\log_{k}n-|S|))$.

Table 1 summarizes the algorithms’ logic and their time complexities.

**Table 1:** Time complexity of algorithms *BBSkConstrained* and *SkylineX.*

Algorithm	BBSkConstrained
**Ordering criterion**	Minimal distance to (0,0)
**Pruning logic**	Dominance relationships with siblings and skyline points
**Worst-case complexity**	$O\;\left(|S{|}^{2}\cdot k\cdot (\log_{k}n-|S|)\cdot \log_{2}\;(|S|\cdot k\cdot (\log_{k}n-|S|))\right)$
**Best-case complexity**	$O\;\left(|S|\cdot (\log_{k}n+|S|)\cdot \log_{2}(\log_{k}n+|S|)\right)$
**Average-case complexity**	$O\;\left(|S|\cdot (k\cdot \log_{k}n+|S|)\cdot \log_{2}(k\cdot \log_{k}n+|S|)\right)$
**Algorithm**	**SkylineX**
**Ordering criterion**	*x*-axis to the origin (0,0)
**Pruning logic**	Updated of range *R*
**Worst-case complexity**	$O(|S|\cdot k\cdot (\log_{k}n-|S|)\cdot \log_{2}(|S|\cdot k\cdot (\log_{k}n-|S|)))$
**Best-case complexity**	$O((\log_{k}n+|S|)\cdot \log_{2}(\log_{k}n+|S|))$
**Average-case complexity**	$O(|S|\cdot k\cdot (\log_{k}n-|S|)\cdot \log_{2}(|S|\cdot k\cdot (\log_{k}n-|S|)))$

## 4. Algorithm for enumerating query

Algorithm 3 calculates the *Enumerating Query* variant on indexed data in a *k*^2^-tree. It uses *BBSkConstrained* over the entire dataset to get the full *Skyline*. For each point found, the number of points that it dominates is efficiently counted using the algorithm in [25], called *Compact Count*.

**Fig 7:**
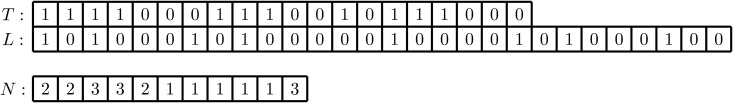
Example of an array *N* for the run example in Fig 4. If *T*(*i*)=1, then the number of points in submatrix *i* is stored in *N*(*rank*_1_(*T*,*i*)).

It is expected that a submatrix is dominated by more than one Skyline point. Therefore, it would be inefficient to count the points twice. We use the Dynamic Programming approach to store the points in every internal node of the tree. The values are ordered in the same way in which the *k*^2^-tree is stored in bitmap *T*. An example of its use is illustrated in Fig 7.

**Algorithm 3. CompactEnumerating(*k*)**

**Input:** *K* The *k*^2^-tree structure

**Output:** The Enumerating Query answer

1: S=∅

2: Insert *root* in *heap*

3: **while** heap⧸=∅ **do**

4:  η= *heap* top

5:  **if** η is not dominated by *S* **then**

6:   **for** each child $\eta_{i}$ in *n* **do**

7:    **if** $\eta_{i}\text{≠}\emptyset \text{ and }\eta_{i}$ is not dominated by *S* **then**

8:     **if** $\eta_{i}$ is not dominated by its non-empty sibling **then**

9:      **if** $\eta_{i}$ is not a leaf **then**

10:       $heap=heap\cup \eta_{i}$

11:      **else**

12:       $r=[x_{\eta_{i}},n-1]\times [y_{\eta_{i}},n-1]$

13:       $num_{\eta_{i}}=CompactCount(k,r)-1$

14:       $S=S\cup (\eta_{i},num_{\eta_{i}})$

15:     **else**

16:      **if** *N*[*rank*_1_ (*k*.*T*, *i*)] is not calculated **then**

17:       *N*[*rank*_1_ (*k*.*T*, *i*)]=*getN*(*k*.*T*, *k*.*L*, *i*)

18: **return** *S*

Values for an array *N* are calculated during the execution because it is not necessary to count the points of all the submatrices. Lines 7 and 8 (Algorithm 3) express the application of the discard conditions. Given that there is no range associated with this query and it is not necessary to apply the fourth discard condition and the precondition of the third discard condition. Line 13 shows the calculation of the number of points that dominate the already identified *Skyline* point using *CompactCount*.

This algorithm is simply a top to bottom Dynamic Programming approach to *Compact Count* [25]; every node calculated is stored in an array *N* and if it needs to be counted again, it is first checked in *N*. Line 16 provides a new situation to precalculate a value in array *N* by counting the number of points within a node dominated by some of its empty siblings. The function *getN()*, defined in [25], calculates the number of points in node *i*. These nodes belong to the dominance region of a *Skyline* point; eventually, the number of points they contain is required.

To explain the algorithm, we will use the points represented in Fig 4. For each outer cycle (*while*) in Algorithm 3.

In the graphics, the points in the Skyline are circled and labeled with the number of points they dominate. The area dominated by the Skyline set is represented by a dotted polygon, and a discard by domination is indicated by a × symbol. Additionally, the array *N* is shown below the matrix. Finally, all nodes involved in the current *while* cycle are highlighted.

**Fig 8:**
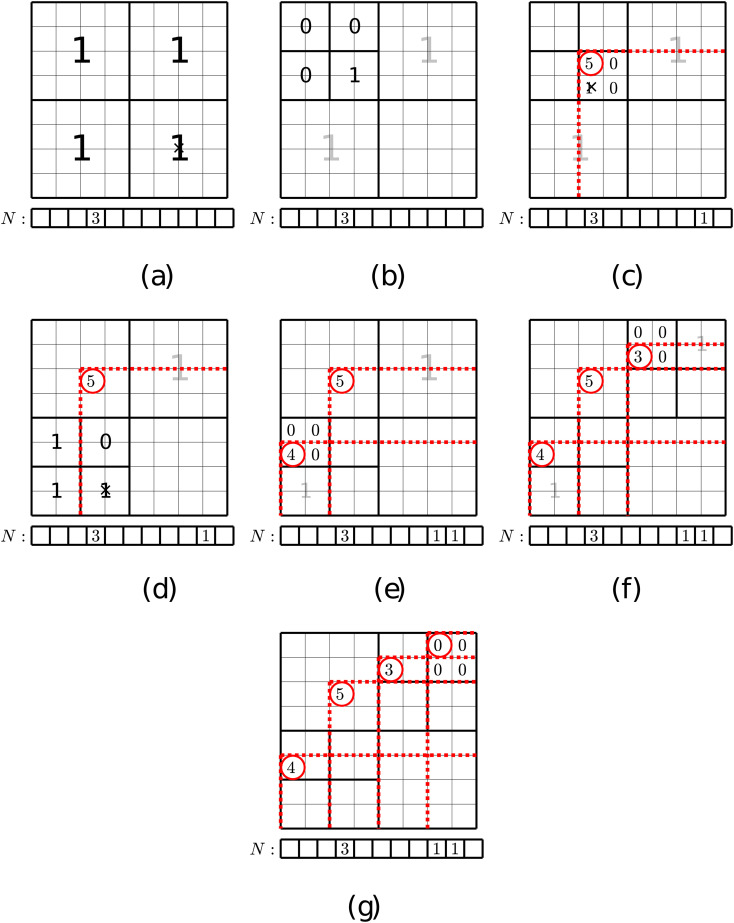
*Compact Enumerating* running example. (a) Checking the root matrix. (b) Checking the first non-empty child. (c) Finding the first point. (d) Counting the points in a dominated submatrix. (e) Finding a second point. (f) Finding a third point. (g) Finding a fourth point.

**Example 3.** *The algorithm starts similarly to Algorithm 1. Therefore, every node in level 1 is inserted into the heap, except for the bottom left node, which is dominated by its upper left sibling (Line 3). Since N is empty (Line 16), it is calculated as N[3] = 3 (see* Fig 8(a)*). The first element of the heap is the upper left node in level 1, which has only one child, as shown in* Fig 8(b)*. Since S is empty (Line 7) and the node has no siblings (Line 8), this only child is inserted into the heap.*

*Now, there are three elements in the heap: the upper right and bottom left nodes in level 1, and the recently added node in level 2 (see* Fig 8(c)*). The node with the lowest MINDIST() is popped first. Its first leaf, located at position (2,2), is not discarded by any condition in lines 7 and 8, because S is empty and its siblings do not dominate it. Being a leaf, it is added to the Skyline set, and the number of points it dominates is counted, as shown in Line 13 (i.e., how many points lie inside the dotted region). The bottom right node in level 1 is already in N; therefore, it must count (and store) the bottom region of the upper right node in level 1 (two submatrices, both empty), plus the right region of the bottom left node in level 1 (two additional submatrices, the first of which is also empty), plus its only sibling, totaling 5 dominated points (see* Fig 4*). Afterward, its only sibling is discarded because it is dominated by the new Skyline point.*

*In this cycle, the upper right and bottom left nodes in level 1 have the same MINDIST() (see* Fig 8(d)*). First, let us examine the bottom left node. It has three non-empty children, two of which intersect with the range; these are added to the heap. The node at the bottom right is discarded in Line 7 because it is dominated by our Skyline point. The upper left child of the bottom left node in level 1 has only one child, which is not discarded under any condition, and it is added to S (see* Fig 8(e)*). The points dominated by this new point are counted in Line 13, which includes the points in the fifth row and all submatrices in the bottom part of level 2 (see* Fig 4*), totaling four points. The value of the newly counted submatrix (from the bottom leftmost node in level 2) is added to N.*

*Next, the upper right node in level 1 is popped from the heap. It has two non-empty children, which are the two nodes at the top (see* Fig 8(f)*). Neither node is dominated by S or its siblings, so both are added to the heap. The leftmost of these two children is now popped from the heap, and it has only one child, which is not dominated by any point in S; thus, it is added to S. We then count the points it dominates, including the points on the right in the second row (all 0), the two empty nodes discarded in the previous step, and the bottom right node in level 1. The last node contains three points, and this value is stored in N. Now, two elements remain in the heap: the bottom leftmost and upper rightmost nodes in level 2 (see* Fig 8(g)*). Starting with the bottom leftmost node, it is immediately discarded by Line 7. The remaining node in the heap has only one child, which is not dominated by anything, so it is added to S. This point dominates all the rightmost nodes in level 2, which are all empty, so it dominates 0 points.*

### 4.1. Theoretical analysis

The temporal analysis of this algorithm is straightforward based on all the results that have already been calculated. Since every node is counted once, it is possible to obtain *N* in time $O(rank_{1}(T,|T|-1))$. However, for each point s∈S, the algorithm still needs to identify the upper nodes dominated by *s* (Line 13). These nodes are called *Maximal Quadrants* in [25], which are the nodes completely inside the dominated area, while their parent is not. As reported in [25], a good average case for the number of maximal quadrants inside an area *r* is $O(\log_{k}r)$.

The best-case scenario for Algorithm 1 is $O(|S|\cdot (\log_{k}n+|S|)\cdot \log_{2}(\log_{k}n+|S|))$. In this case, only the first Skyline points have a height equivalent to the tree height, and the rest are near the bottom. We can therefore assume that there is a constant number of count queries in the higher parts of the tree. Accordingly, *r* is *O*(*n*^2^), and the best-case scenario for Algorithm 3 is $O(((|S|+\log_{k}n)\log_{k}n+|S{|}^{2})\cdot \log_{2}(\log_{k}n+|S|))$.

For the worst-case scenario, we consider Algorithm 1: $O(|S{|}^{2}\cdot k\cdot (\log_{k}n-|S|)\cdot \log_{2}(|S|\cdot k\cdot (\log_{k}n-|S|)))$. We assume a fraction of the Skyline points have a height in the higher part of the tree. In these cases, *r* is again *O*(*n*^2^), and we need to count almost every internal node, so the count is O(m·logn), with *m* the amount of points. Therefore, the worst-case scenario for Algorithm 3 is $O(|S{|}^{2}\cdot k\cdot (\log_{k}n-|S|)\cdot \log_{2}(|S|\cdot k\cdot (\log_{k}n-|S|))+m\cdot \log n)$.

Similarly, the average case would need to count a fraction of the internal nodes with an asymptotic behavior of $O(|S|\cdot (k\cdot \log_{k}n+|S|)\cdot \log_{2}(k\cdot \log_{k}n+|S|)+m\cdot \log n)$.

Table 2 summarizes the algorithm’s logic and its time complexity.

**Table 2:** Time complexity of algorithm *Compact Enumerating.*

Algorithm	Compact Enumerating
**Ordering criterion**	Minimal distance to (0,0)
**Pruning logic**	Dominance relationships with siblings and skyline points
**Count logic**	Compact count of dominated points [25]
**Worst-case complexity**	$O(|S{|}^{2}\cdot k\cdot (\log_{k}n-|S|)\cdot \log_{2}(|S|\cdot k\cdot (\log_{k}n-|S|))+m\cdot \log n)$
**Best-case complexity**	$O(((|S|+\log_{k}n)\log_{k}n+|S{|}^{2})\cdot \log_{2}(\log_{k}n+|S|))$
**Average-case complexity**	$O(|S|\cdot (k\cdot \log_{k}n+|S|)\cdot \log_{2}(k\cdot \log_{k}n+|S|)+m\cdot \log n)$

## 5. Experimentation

A set of experiments is presented in this section with the aim of evaluating the performance of the algorithms measured as execution time and the amount of memory used (peak memory utilization).

The implemented algorithms were tested on a server with four Intel (R) Xeon (R) CPU E3-1225 processors with 3.30GHz and 8192 KB cache memory. For time measurement, we used the <chrono> library available in C/C++. For memory measurement, we used the command shell script /usr/bin/time; its -f option allows us obtain the peak memory utilization of each process. The resources used during the data input/output were not considered.

We consider both, synthetic and real datasets. For the former, two types of distribution were considered, namely uniform and clustered. Set sizes of 0.1M, 0.5M, 1M, 5M, 10M, and 50M points were also studied. Ten datasets were generated for each configuration (number of points, matrix size, and distribution), and the algorithms were run 10 times for each set. Therefore, the result for each configuration was the average of 100 runs. The standard deviations were negligible relative to the reported execution times; therefore, they are omitted from the figures. For the latter, real datasets represent a set of twelve monthly snapshots of Web graphs over .uk domains, which were obtained between June 2006 and May 2007 from the Laboratory for Web Algorithmic (https://law.di.unimi.it/). Table 6 presents the number of points and the density for each snapshot. Each snapshot *i* can be interpreted as a binary relation $R_{i}$ over the set *N*, where *N* denotes the nodes of the Web graph, specifically, the web pages within the .uk domain. In this context, a relation $p\;R_{i}\;q$ holds if page *p* contains a hyperlink to page *q*. These datasets were used in [26] to experimentally evaluate set operation algorithms over compact data structures (All code developed in this work is publicly available in the GitLab repository (https://gitlab.com/mmunocan/skyline), and the datasets can be accessed via Figshare (https://doi.org/10.6084/m9.figshare.32118016)).

### 5.1. Skyline Query

Although the aim of this article is to contribute algorithms to evaluate the Constrained and Enumerating Skyline Query variants, the *Skyline Query* is evaluated in this section as a special case of the Constrained Skyline Query in which the range query is the matrix size. Synthetic data with uniform and clustered distributions were included in the evaluation. We consider two matrix sizes: 65,536×65,536 and 1,048,576×1,048,576.

The algorithms in the charts are identified as *BBSK Manhattan* and *BBSK Euclidean* to refer to the *BBSkConstrained* (Algorithm 1) algorithm using the Manhattan distance and Euclidean distance, respectively, and *SkylineX* to refer to the *SkylineX* (Algorithm 2) algorithm.

**Fig 9:**
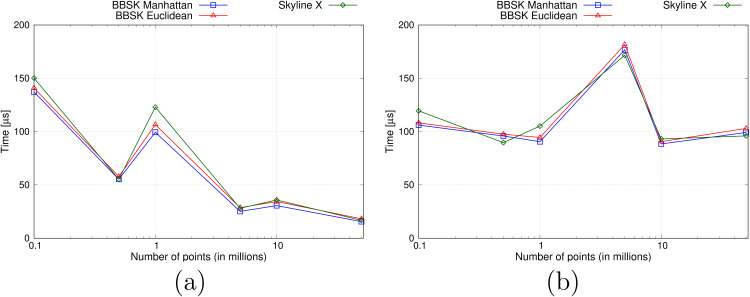
Execution time Skyline Query. Execution time (in μs) over Skyline Query versus the number of points (the *x*-axis is on a logarithmic scale) considering a matrix size of 65,536×65,536. (a) Uniform data. (b) Clustered data.

Fig 9 shows the execution time of the algorithms compared with the number of points over datasets of different sizes with uniform and clustered distributions considering a matrix size of 65,536×65,536. There are no significant differences between *BBSK Manhattan*, *BBSK Euclidean*, and *SkylineX* in which the time used is approximately 100 μs.

**Fig 10:**
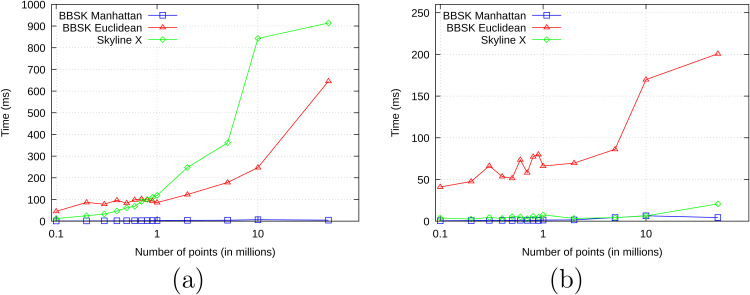
Execution time Skyline Query over a large matrix size. Execution time (in *ms*) over Skyline Query versus the number of points (the *x*-axis is on a logarithmic scale) considering a matrix size of 1,048,576×1,048,576. (a) Uniform data. (b) Clustered data.

Fig 10 presents the execution times for a matrix of size 1,048,576×1,048,576. Under the uniform distribution, the *BBSK Manhattan* algorithm demonstrates the best performance, while the *SkylineX* algorithm exhibits the poorest performance, with a difference of approximately two orders of magnitude. This difference can be explained by the theoretical analysis, which shows that *SkylineX* is expected to operate near its worst-case scenario. Its performance improves only depending on the order and positions of the skyline points identified by the algorithm. In contrast, under a clustered data distribution, both *BBSK Manhattan* and *SkylineX* achieve similarly high performance, illustrating a favorable order and positioning of the skyline points.

**Fig 11:**
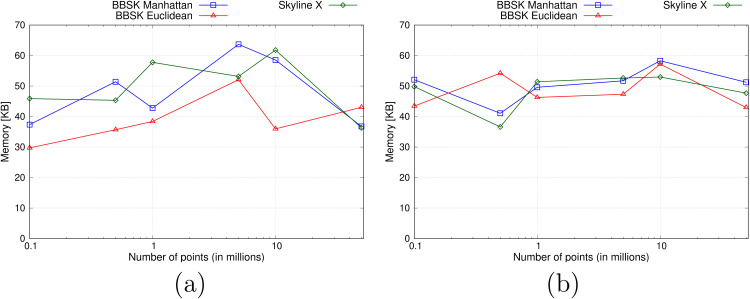
Memory peak of Skyline Query. Memory peak (in KB) over Skyline Query versus the number of points (the *x*-axis is on a logarithmic scale) considering a matrix size of 65,536×65,536. (a) Uniform data. (b) Clustered data.

Fig 11 shows the memory peak utilization of the algorithms compared with the number of points over different data distributions considering a matrix size of 65,536×65,536. As with execution time, there were no major differences in memory peaks between algorithms and between distributions; the maximum was approximately 65 KB, which is a very small amount of additional memory consumed by the algorithms.

**Fig 12:**
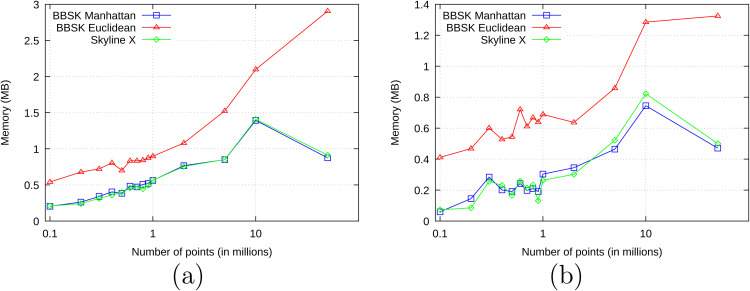
Memory peak of Skyline Query over a large matrix size. Memory peak (in MB) over Skyline Query versus the number of points (the *x*-axis is on a logarithmic scale) considering a matrix size of 1,048,576×1,048,576. (a) Uniform data. (b) Clustered data.

Fig 12 illustrates the peak memory utilization of the algorithms in relation to the number of points across different data distributions, considering a matrix size of 1,048,576×1,048,576. In this case, no significant differences were observed among most of the algorithms. However, the *BBSK Euclidean* algorithm exhibits inferior performance, requiring approximately twice the amount of memory compared to the others.

### 5.2. Constrained Skyline Query

The *BBSkConstrained* algorithms are evaluated in this section (Algorithm 1) and *SkylineX* (Algorithm 2). Eight different range query sizes were considered, namely 0.01%, 0.1%, 1%, 10%, 25%, 50%, 99%, and 100% of the matrix size. The ranges included sides that were parallel to the axes and located in the center of the matrix. The algorithms were described in the graphs as follows. For the *BBSkConstrained* algorithm, *BBSK Constrained Manhattan* allowed us to identify the variant that uses the Manhattan distance and *BBSK Constrained Euclidean* to identify the Euclidean distance. As for the *SkylineX* algorithm, *Constrained X* was used to identify it.

Uniform and clustered distributions were included in all the experiments with synthetic data. However, results were similar for both distributions; therefore only the results of the experiments over sets with uniform distribution are shown and discussed in this and the next sections. The results for this distribution are included because it is the least favorable for the *k*^2^-tree.

**Fig 13:**
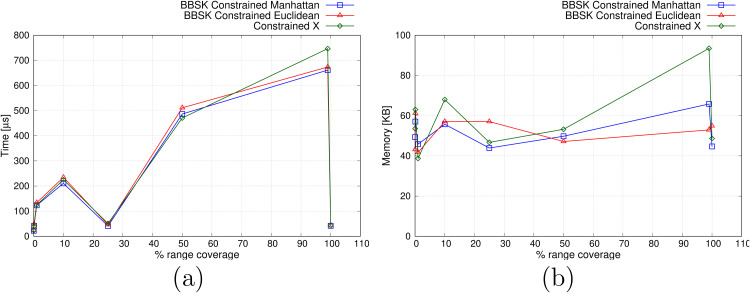
Performance of Constrained Skyline Query. Performance of Constrained Skyline Query versus range query size. (a) Execution time (in μs). (b) Memory peak (in KB).

Fig 13 shows the resources used by the algorithms compared with the range query size. These experiments involved a matrix size of 65,536×65,536 with 1M points or 1M cells in 1. For execution time (Fig 13(a)), the three algorithms exhibited a similar performance. Overall, as the range size increased, the performance of the three algorithms worsened, and the worst performance occurred with a 99% range. However, the best performance occurred with the 100% range, which is equivalent to what is illustrated in Fig 9(a). This is because the number of Maximal Quadrants to explore is reduced to 1, which is the complete matrix. Something similar occurs in the 25% range. In contrast, the 99% range resembles the worst-case scenario. The differences between the three algorithms were not significant for the memory peak (Fig 13(b)). The behavior of the execution time was repeated for the 99% and 100% ranges.

**Fig 14:**
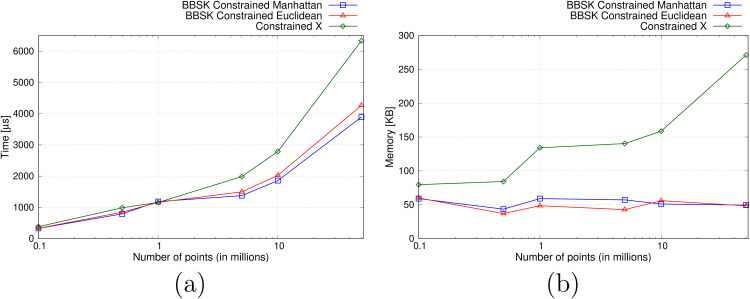
Performance of Constrained Skyline Query. Performance of Constrained Skyline Query versus number of points (the *x*-axis is on a logarithmic scale). (a) Execution time (in μs). (b) Memory peak (in KB).

Fig 14 shows the performance of the algorithms with a matrix size of 65,536×65,536, set size (number of points) of 0.1M, 0.5M, 1M, 5M, 10M, and 50M, and range size of 99% of the matrix size. From sets with a 5M size, *BBSK Constrained Manhattan* is approximately 1.4 times faster than *SkylineX* and requires less than 0.3 of the additional memory consumed by the latter.

**Fig 15:**
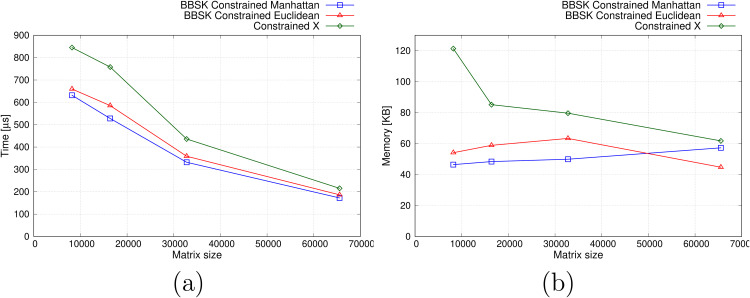
Performance of Constrained Skyline Query versus matrix size. (a) Execution time (in μs). (b) Memory peak (in KB).

Fig 15 shows the resources used by the algorithms compared with the matrix size. These experiments consisted of 1M size sets, query range of [1,8191]×[1,8191], and a matrix size varying between 8,192×8,192, 16,384×16,384, 32,768×32,768, and 65,536×65,536. The three algorithms improved their execution time as the matrix size increased. In addition, *BBSK Constrained Manhattan* and *BBSK Constrained Euclidean* slightly outperformed *Constrained X*. Regarding the memory peak, *Constrained X* was affected by the matrix size and usually consumed slightly more memory than the other two algorithms.

### 5.3. Enumerating Skyline Query

We compare our *Compact Enumerating* (Algorithm 3) algorithm with the following two algorithms. The first is *Range Enumerating* that is a solution for the Enumerating Skyline Query in which *BBSkConstrained* is used over the set of points to obtain Skyline points, and *Range* [27] is then used to count the dominated points. The second is *Range Compact Enumerating* that is a solution for the Enumerating Skyline Query in which *BBSkConstrained* is used over the set of points to obtain Skyline points, *Compact Count* [25] is then used to count the dominated points.

**Fig 16:**
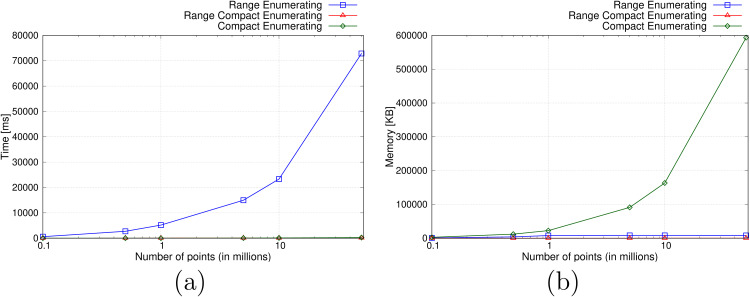
Performance of enumerating Skyline Query. Performance of Enumerating Skyline Query versus number of points (the *x*-axis is on a logarithmic scale) considering a matrix size of 65,536×65,536. (a) Execution time (in *ms*). (b) Memory peak (in KB).

Fig 16 shows the resources used by the algorithms compared with the number of points. The experiments involved a matrix size of 65,536×65,536 and set sizes of 0.1M, 0.5M, 1M, 5M, 10M, and 50M. For execution time, *Range Enumerating* was the algorithm that used the most time, on the order of seconds. For memory used, *Compact Enumerating* was the algorithm that consumed the most.

**Fig 17:**
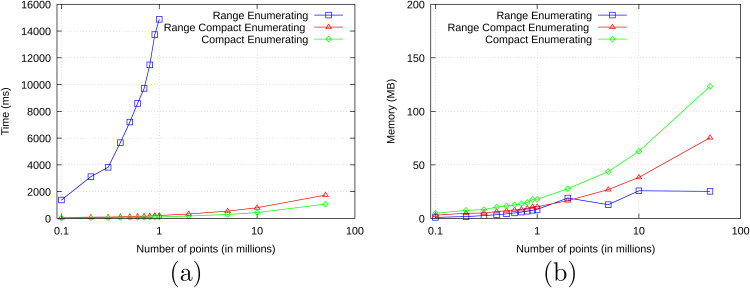
Performance of enumerating Skyline Query on a large matrix size. Performance of Enumerating Skyline Query versus number of points (the *x*-axis is on a logarithmic scale) considering a matrix size of 1,048,576×1,048,576. (Execution time (in *ms*)). (b) Memory peak (in KB).

Fig 17 presents the resource consumption of the algorithms as a function of the number of points. The experiments were conducted on a matrix of size 1,048,576×1,048,576, with dataset sizes of 0.1M, 0.5M, 1M, 5M, 10M, and 50M points. Among the three algorithms, the *Compact Enumerating* algorithm achieves the best execution time, while the *Range Enumerating* algorithm shows the poorest performance, with a difference of at least two orders of magnitude. Regarding memory consumption, all algorithms demonstrate competitive behavior, with the *Range Enumerating* algorithm achieving the lowest memory usage.

Therefore, the most competitive algorithm of this analysis was *Range Compact Enumerating*, which had a good performance in terms of both time and memory. The behavior of *Compact Enumerating* is due to the arrangement used to maintain the relations or points counted in the visited submatrices.

### 5.4. Comparison between *BBSkConstrained* algorithm with the *BBS* on R-tree

In this section, we present experimental results comparing the performance of the *BBSkConstrained* algorithm with that of the *BBS* algorithm proposed in [28,29], which is based on the R-tree structure. We evaluate *BBSkConstrained* using the Manhattan distance metric, as it represents the best-performing variant of our approach. In contrast, *BBS* is implemented using the Euclidean distance metric, as this configuration yields its best performance.

The experiments were conducted on datasets ranging in size from 100,000–50,000,000 points in a two-dimensional space, defined over a domain of 10,000,000×10,000,000. To evaluate scenarios with higher densities in main memory for the R-tree, a smaller domain of 4,096×4,096 was also considered, with point densities of 1%, 5%, and 10%. Both uniformly distributed and clustered datasets were included in the evaluation.

Each dataset was indexed using three structures: an adjacency list, a *k*^2^-tree, and an R-tree (including both index and data). Table 3 reports the storage requirements as a function of the number of points and the density. All reported values correspond to the average over ten datasets generated for each configuration.

**Table 3:** File Sizes (in KB) for Synthetic Data. Size ratio is the ratio between R-tree divided by k^2^-tree.

	Uniform Distribution				Clustered Distribution			
# of	Size [KB]			Size	Size [KB]			Size
points	Plain	k^2^-tree	R-Tree	ratio	Plain	k^2^-tree	R-Tree	ratio
100,000	1,540	756	7,037	9.31	1,549	731	7,044	9.64
200,000	3,081	1,461	14,061	9.62	3,089	1,403	14,083	10.04
300,000	4,622	2,147	21,080	9.82	4,656	2,057	21,103	10.26
400,000	6,163	2,820	28,150	9.98	6,189	2,709	28,154	10.39
500,000	7,704	3,485	35,192	10.10	7,708	3,319	35,199	10.61
600,000	9,244	4,141	42,227	10.20	9,249	3,888	42,241	10.86
700,000	10,785	4,791	49,259	10.28	10,775	4,533	49,261	10.87
800,000	12,326	5,436	56,288	10.35	12,403	5,175	56,329	10.88
900000	13,867	6,077	63,308	10.42	13,905	5,836	63,348	10.85
1,000,000	15,407	6,713	70,367	10.48	15,378	6,378	70,374	11.03
2,000,000	30,815	12,913	140,732	10.90	30,971	12,256	140,793	11.49
5,000,000	77,040	30,587	351,870	11.50	77,056	29,019	351,986	12.13
10,000,000	154,079	58,612	703,696	12.01	155,034	54,908	704,177	12.82
50,000,000	770,399	263,298	3,519,115	13.37	772,216	251,251	3,520,639	14.01
**Density**								
1%	1,549	270	11,797	43.69	1,568	225	11,806	52.47
5%	7,747	874	59,004	67.51	7,861	625	59,118	94.59
10%	15,495	1,356	118,119	87.11	15,533	1,010	118,190	117.02

As shown in Table 3, in terms of storage requirements, the R-tree requires between 9 and 14 times more space than our approach.

Table 4 presents the execution time of *BBSkConstrained* (using the Manhattan distance) compared with *BBS* (using the Euclidean distance) under two scenarios: (i) when the R-tree is fully stored in main memory, and (ii) when the R-tree resides in secondary memory. In scenario (i), the results strongly favor *BBS*, which outperforms our approach by a factor ranging from 32 to 113 in the case of uniformly distributed data, and from 11 to 43 for clustered distributions. In contrast, in scenario (ii), our approach significantly outperforms *BBS*, achieving improvements of up to five orders of magnitude.

**Table 4:** Execution times (in *ms*) comparing *BBSkConstrained* using the Manhattan distance and *BBS* based on the R-tree using the Euclidean distance, as a function of the number of points.

Uniform D.	Times [ms]			Speedup
# points	BBSk	BBS M.M.	BBS S.M.	ratio
100,000	0.97	0.03	347.50	32.33
200,000	1.29	0.04	848.40	32.25
300,000	1.59	0.04	1,512.30	39.75
400,000	1.84	0.04	1,994.55	46.00
500,000	2.11	0.04	2,494.93	52.75
600,000	2.00	0.04	3,905.33	50.00
700,000	2.39	0.04	4,353.68	59.75
800,000	2.49	0.03	4,306.91	83.00
900,000	2.59	0.04	5,842.94	64.75
1,000,000	2.98	0.04	6,570.08	74.50
2,000,000	3.23	0.03	12,272.84	107.67
5,000,000	3.42	0.04	34,040.61	85.50
10,000,000	4.40	0.05	103,304.92	88.00
50,000,000	5.66	0.05	497,504.07	113.20
**Clustered D.**				
100,000	0.46	0.04	345.99	11.50
200,000	0.72	0.06	979.83	12.00
300,000	0.57	0.05	1,514.33	11.40
400,000	0.87	0.07	2,446.30	12.43
500,000	1.07	0.06	2,944.30	17.83
600,000	1.15	0.06	3,550.44	19.17
700,000	1.35	0.07	4,838.28	19.29
800,000	0.88	0.04	4,109.87	22.00
900,000	1.48	0.05	5,770.25	29.60
1,000,000	1.58	0.08	7,836.72	19.75
2,000,000	1.27	0.05	13,246.49	25.40
5,000,000	1.94	0.06	41,937.88	32.33
10,000,000	2.07	0.07	114,187.66	29.57
50,000,000	3.47	0.08	599,104.07	43.38

Table 5 reports the execution time of *BBSkConstrained* (using the Manhattan distance) compared with *BBS* (using the Euclidean distance) under the same two scenarios as before: (i) when the R-tree is fully stored in main memory, and (ii) when the R-tree resides in secondary memory, now considering higher data densities. In scenario (i), execution times are comparable for both uniform and clustered distributions. This behavior is expected, as the running time of *BBSkConstrained* depends on the logarithm of the domain size and the size of the output, rather than directly on the number of points. Nevertheless, in scenario (ii), our approach continues to significantly outperform *BBS*, achieving improvements of up to five orders of magnitude.

**Table 5:** Execution times (in *ms*) comparing *BBSkConstrained* using the Manhattan distance and *BBS* based on the R-tree using the Euclidean distance, considering datasets with higher densities.

Uniform D.	Times [ms]			Speedup
# points	BBSk	BBS M.M.	BBS S.M.	ratio
1%	0.04	0.02	214.56	2.00
5%	0.02	0.02	1,038.22	1.00
10%	0.01	0.02	2,318.04	0.50
**Clustered D.**				
1%	0.06	0.03	373.60	2.00
5%	0.08	0.05	3,505.35	1.60
10%	0.05	0.02	4,291.65	2.50

These results are consistent with theoretical expectations. In scenario (i), the observed behavior is anticipated, as the higher computational cost of *BBSkConstrained* compared to *BBS* at lower densities can be attributed to the low arity of the *k*^2^-tree and the overhead associated with processing compressed data. In contrast, in scenario (ii), the higher execution time of *BBS* relative to *BBSkConstrained* is primarily due to the cost of I/O operations.

In summary, the experimental results indicate that our approach is well suited for deployment on devices with limited computational resources, particularly with respect to main memory usage. In contrast, the memory overhead required to maintain the entire R-tree in main memory is prohibitive.

### 5.5. Real datasets

Table 6 (Dataset Description) provides details of the datasets used in the experimentation. The second column indicates the number of points, relations, or cells in 1. The third column represents density, that is, the percentage of 1s compared with the total number of cells in the matrix, (1) is *Range Enumerating*, (2) corresponds to *Range Compact Enumerating*, and (3) is *Compact Enumerating*, *v*_1_ and *v*_2_ are acceleration metrics defined by $v_{1}=\frac{Range\;Enum.}{Range\;Compact}$ and $v_{2}=\frac{Range\;Enum.}{Compact\;Enum.}$ All the sets were stored in a *k*^2^-tree with a matrix size of 1,000,000×1,000,000.

According Table 6-(a) the *Range Compact Enumerating* and *Compact Enumerating* algorithms outperform the *Range Enumerating* algorithm when evaluating the Enumerating Skyline Query. Specifically, *Range Compact Enumerating* is on the average 543 times faster than *Range Compact Enumerating* and 417 times faster than *Compact Enumerating*.

**Table 6:** Results for Enumerating Skyline Query over real datasets. (a) Execution time (in μs) and (b) Memory peak (in KB).

(a) Execution time (in *µ*s)							
Dataset Description			Execution time (*µ*s)			Acceleration	
Id	#Points	Density					
Dataset	(#1s)	(%)	(1)	(2)	(3)	*v*_1_	*v*_2_
snap00	2,404,620	0.00024	909,514	1,981	3,004	459	302
snap01	1,360,019	0.00014	534,874	1,762	3,336	303	160
snap02	3,200,913	0.00032	1,362,632	2,466	2,200	552	619
snap03	3,395,695	0.00034	1,634,348	1,613	2,194	1,013	745
snap04	2,350,289	0.00024	1,101,352	1,532	1,523	719	723
snap05	2,526,547	0.00025	1,074,464	1,862	2,060	577	521
snap06	1,507,033	0.00015	550,513	1,462	2,137	376	257
snap07	1,950,981	0.00020	689,645	1,906	1,568	361	439
snap08	1,772,026	0.00018	159,631	349	1,445	457	110
snap10	2,665,612	0.00027	465,500	698	923	667	504
snap11	1,989,568	0.00020	520,716	1,042	2,500	499	208
**(b) Memory Peak**							
**Id**							
**Dataset**	**(1)**	**(2)**	**(3)**				
snap00	8,181	34	10,837				
snap01	8,189	47	6,151				
snap02	8,186	38	1,5873				
snap03	8,209	47	19,203				
snap04	8,185	43	12,693				
snap05	8,183	38	12,468				
snap06	8,181	43	6,380				
snap07	8,183	47	8,034				
snap08	8,186	46	7,544				
snap10	8,190	49	11,067				
snap11	8,204	42	8,231				

Table 6-(b) indicates the memory peak of the Enumerating Skyline Query over the real datasets of (1) *Range Enumerating*, (2) *Range Compact Enumerating*, and (3) *Compact Enumerating*. *Range Compact Enumerating* is the proposal that uses the least additional memory, while *Compact Enumerating* requires the most memory. These results are consistent with the findings in Section 5.3 for synthetic datasets.

**Fig 18:**
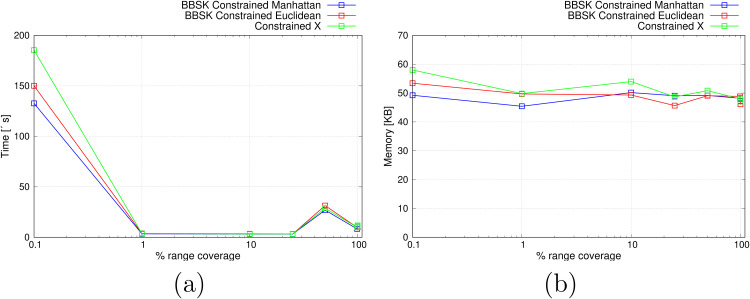
Performance of Constrained Skyline Query over real datasets. Performance of Constrained Skyline Query over real datasets (the *x*-axis is on a logarithmic scale). (a) Execution time (in μs). (b) Memory peak (in KB).

Fig 18 illustrates the resources used in the Constrained Skyline Query over real datasets. The range queries are 0.01%, 0.1%, 1%, 10%, 25%, 50%, 99%, and 100%. The ranges include sides that are parallel to the axes and located in the center of the matrix. The charts use the same labels as those in Section 5.2 to refer to the algorithms. As with the synthetic data, there were no significant differences between the algorithms. For time measurement, it is noteworthy that the peak for all algorithms occurred in the 0.1% range query, which was even higher than in the worst-case scenario for the 99% range. The memory peak also remained relatively constant for all algorithms, regardless of the range query.

## 6. Conclusions

This paper proposes algorithms to calculate two variants of the *Skyline Query* over a set of points stored in the *k*^2^-tree compact data structure. Specifically, algorithms *BBSkConstrained* and *SkylineX* are proposed for the *Constrained Skyline* variant and algorithm *Compact Enumerating* for the *Enumerating Skyline* query. The algorithms take advantage of the *k*^2^-tree properties, and they are solved by accessing only a small portion of the dataset.

The experimental results confirm that our algorithms achieve more efficient resource management than the naive approach. In particular, *BBSkConstrained*, when using the Manhattan distance, outperforms *SkylineX*. This improvement can be attributed to its ability to prune a larger number of nodes at earlier stages, as the initial skyline points are closer to the origin.

Our algorithms enable the direct computation of the *Skyline Query* over datasets represented using the compact *k*^2^-tree structure. Previously, the only available approach to obtain skyline points from such data required full decompression followed by the application of a non-indexed algorithm. As demonstrated by the experimental evaluation, this decompression step can be prohibitive in terms of main memory consumption.

As future work, we aim to explore the implementation of a novel approach that approximates the solution set by selecting a representative subset, as proposed in [30]. Additionally, we plan to extend our current work by increasing the number of dimensions considered in the Skyline Query, thereby enabling more complex multi-criteria analysis, This will enable the method to be applied across a wide range of domains. For example, it can be used in recommender systems and multicriteria decision-making systems, among others. In many such applications, it is necessary to solve skyline problems in three or more dimensions.

The broader applicability of queries over compact data structures lies in their potential to enable fully compacted databases instead of raw representations. However, the feasibility of such an approach depends on the availability and efficiency of essential query operations over compact representations. Therefore, further research is needed to expand the repertoire of supported queries and to design more versatile compact data structures capable of handling diverse data types, including high-dimensional data, raster datasets, polygons, and other complex spatial representations.
