# Supplementary material for: Computing the Skyline Query over a k2-tree compact data structure
Source: PLoS One. 2026 Jul 17;21(7):e0353358. doi: 10.1371/journal.pone.0353358 (PMC13379023; doi:10.1371/journal.pone.0353358)
Supplement: Attachment — Submitted filename: answers to reviewers(v2).pdf [file pone.0353358.s001.pdf]

Answers to Reviewers

Please use the space provided to explain your answers to the questions above. You may also include additional comments for the author, including concerns about dual publication, research ethics, or publication ethics. (Please upload your review as an attachment if it exceeds 20,000 characters)

Reviewer #1: The paper effectively outlines the problem and proposes novel algorithms to perform Skyline Queries directly on the k2-tree compact data structure. This innovation is clearly articulated, providing a strong foundation for the reader. However, more clarity could be provided on the specific computational benefits of the k2-tree in comparison to other compact data structures (e.g., quadtree or R-tree) in Skyline Query applications.

1. The claim of being the first implementation of Skyline Queries on compact structures **needs stronger evidence and a comparative literature table**.

We modify the statement in the introduction by: This paper builds upon the work presented in \cite{TGMC2024} by offering more detailed explanations of the algorithms, expanding the discussion of the theoretical results, and presenting additional experimental analyses, including a comparison of skyline query computation using R-tree based structures.

2. Broader impact should be clarified through concrete application domains (e.g., large web-graph analytics, spatial decision support).

We listed applications of the skyline query in the introduction.

3. Quantify the real-world gain (e.g., % memory/time savings) to highlight significance.

We have added a new Section 5.4 where we address this issue.

4. However, Algorithms are clearly structured and theoretically justified with lemmas and complexity analyses. Provide explicit differentiation from prior BBS, R-tree, and bitmap-based Skyline methods.

The prior methods for BBS work with data in secondary memory while our algorithms work on main memory. Therefore, prior methods focalize on input/output operations and our proposal focuses on the number of operations on the CPU. As a conclusion, both methods follow a different approach and it is not possible to theoretically compare them.

We add a phase about this when introducing compact data structures.

5. I would suggest to include a summary table comparing structure type, need for decompression, time complexity, and memory overhead.

As explained before it is not possible to compare prior methods with the methods based on compact data structures.

6. Add brief explanatory comments in pseudocode.

The algorithms are explained in detail in the paragraphs following their introduction. We also describe their execution in Examples 1 and 2.

7. Provide a small, visual example (n = 8) showing how pruning and heap updates occur.

We present Examples 1 and 2, in which we describe their executions in detail.

8. Present a table contrasting BBSk Constrained vs Skyline X (ordering criterion, pruning logic, expected complexity).

We include Table 1 and Table 2 that summarize the algorithms in terms of logic and complexity.

9. Summarize each lemma's conclusion in plain language and include one table listing best, expected and worst-case complexities.

We include Table 1 and Table 2 that summarize the algorithms in terms of logic and complexity.

10. In Experimental Design- Add baseline comparison with a decompressed (plain) Skyline algorithm; Analyze scalability with respect to k and matrix size n.

We added Section 5.4 where we compare our algorithm with the BBS over R-trees.

11. Report standard deviations or error bars over the 10 × 10 runs.

We considered that the standard deviations are negligible with respect to the reported times. So, we do not include them.

12. Add numerical summaries below figures (e.g., “BBSK Manhattan achieved 45 % less memory than Skyline X”).

We present the numerical differences in algorithm execution within the manuscript, where the figures are discussed in detail. Since these differences do not depend solely on the algorithms themselves, including such explanations in the figure captions would make them unnecessarily crowded.

13. Discuss why Skyline X underperforms for uniform distributions yet matches performance for clustered ones.

We added an explanation when we describe Figure 10.

14. Include a brief correctness check/validation against standard Skyline output to confirm identical results.

The theoretical proof of the correctness of the algorithms is included in the manuscript.

15. Add a short “Contributions” paragraph at the end of the Introduction summarizing: First Skyline computation on compact structure, Two Constrained Query variants, Enumerating Query algorithm with complexity proof, and Empirical performance gains.

Done.

16. Ensure consistent symbol usage (η, n, r, S) across pseudocode and text.

Done.

17. Check equation formatting (subscripts/superscripts) and figure captions.

Done.

18. The conclusion is concise, but further exploration into the practical implications of extending Skyline Queries to more than two dimensions could be discussed. Multi-dimensional Skyline Queries are a common challenge in many fields, and expanding the method to handle them efficiently would be a valuable contribution.

Done.

Reviewer #2: The paper proposes algorithms to compute two variants of the Skyline Query—Constrained Skyline Query and Enumerating Skyline—directly on k²-tree compact representations of 2D point sets, without decompression. It builds on the Branch-and-Bound Skyline (BBS) approach, adapting it to k²-tree's recursive structure for efficient dominance checks and pruning. Theoretical analyses and experiments on synthetic and real datasets (Web graphs) demonstrate improved efficiency over non-indexed baselines.
Overall, the manuscript addresses a relevant gap in integrating multi-criteria queries with compact data structures, which is timely given the growth of large-scale spatial data. The k²-tree's application to Skyline Queries is innovative, and the direct computation on compressed data is a strength. However, the claimed "first study" overlooks the authors' own prior conference publication (cited as [7]), which reduces novelty for a journal extension. Methodological details are solid but could be enhanced for reproducibility (e.g., code availability). Clarity is good, though some sections (e.g., theoretical proofs) are dense. I recommend some revisions to address self-citation, expand novelty discussion, improve reproducibility, and refine analyses. Below, I detail major and minor concerns.

Major Concerns

1. Novelty and Relation to Prior Work: The abstract claims this is "the first study to implement Skyline Queries directly over compact data structures, specifically the k²-tree." However, this is an extension of the authors' own conference paper [7] (SCCC 2024), which already introduced these algorithms and experiments. While extensions are acceptable for PLOS ONE, the manuscript must explicitly discuss added contributions (e.g., deeper theoretical analyses, more proofs, expanded experiments) and cite [7] prominently in the abstract/introduction. Currently, [7] is mentioned briefly (p. 2), but the novelty claim is overstated—revise to emphasize journal-specific advancements. Also, discuss related work more broadly (e.g., skyline on other compacts like wavelet trees [web:3 from search], or distance joins on k²-tree).

We now present additional experimental analyses, an improved theoretical analysis, and a comparison of skyline query computation using R-tree based structures.

2. Reproducibility and Data Availability: PLOS ONE requires data/code sharing for verification. The manuscript lacks details on code implementation (e.g., C++ libraries for k^2-tree, heap) or repository links. Synthetic data generation is described vaguely (e.g., uniform/clustered—provide parameters/algorithms). Real datasets are cited (LAW lab), but ensure direct access or deposition (e.g., in Figshare/Zenodo). Add a Data Availability Statement per PLOS guidelines, including scripts for experiments.

We added this information in the experimental section.

All code developed in this work is publicly available in the GitLab repository (https://gitlab.com/mmunocan/skyline), and the datasets can be accessed via Figshare (https://doi.org/10.6084/m9.figshare.32118016).

3. Theoretical Analysis Limitations: Proofs are correct but assume uniform/worst-case distributions without empirical validation (e.g., how often does the expected case O(k · log_k n + |S|) hold?). For Enumerating, the O(m · log n) counting cost (m = points) could dominate for dense datasets—discuss scalability thresholds. Clarify assumptions (e.g., k=2 in experiments; generalize to arbitrary k). Extend to higher dimensions (future work mentions this but suggest feasibility).

When using compact data structures, it is typically assumed that the data are clustered and sparse; otherwise, the structures do not achieve effective compression.

4. Experimental Scope: While comprehensive, add comparisons to index-based baselines (e.g., BBS on R-tree [2]) on decompressed data to quantify compression benefits. Real datasets are sparse (density <0.00034%), favoring k^2-tree—test denser ones (e.g., geospatial points). Report standard deviations for averages (100 runs) to assess variability. Memory peaks are low (~MB), but profile heap vs. N array contributions in Compact Enumerating.

We add Section 5.4 where we report the comparison between our BBSK algorithm with the BBS over R-trees.

In spatial databases it is not common to find dense datasets, since by nature spatial data are sparse. However, we include different levels of density in the experiments in Section 5.4.

We decided to not include the standard deviations since they are negligible with respect to the reported times.

Minor Concerns

1. Clarity and Presentation: Some notations are inconsistent (e.g., "k2-tree" vs. "k^2-tree"; use superscript).

We performed a complete revision of the paper's grammar.

Proofs (e.g., Lemma 4) could use more steps for accessibility.

We re-organized the lemmas and their explanations to better understand. Also, we generate two theorems that allow us to conclude the results of the theoretical analysis of algorithms.

Figures (e.g., 9-18) lack error bars; legends are clear but add units (e.g., μs/ms).

Done.

English is good but polish awkward phrases (e.g., "the query remains relevant in the context of two-dimensional sets" → "remains applicable to 2D sets").

Done.

Add subheadings in sections (e.g., "Algorithm Description" vs. "Theoretical Analysis").

We improved the manuscript by adding an Example environment. Now the lecture is more appropriate.

2. Related Work Gaps: Expand on skyline variants (e.g., dynamic skylines [9], probabilistic) and k^2-tree applications (e.g., graphs [6], set operations [22]). Discuss limitations of 2D focus (e.g., vs. higher-d k^2-raster).

We added more discussion in the Conclusion Section.

Reviewer #3:

Summary

This manuscript presents a clear and rigorous study on implementing and evaluating Skyline Query variants directly over the k^2-tree compact data structure. The authors successfully demonstrate that Constrained Skyline and Enumerating Skyline queries can be efficiently computed without decompressing data, achieving strong performance in both time and memory efficiency. The work bridges two active areas—compact data structures and multi-criteria query processing—and represents the first concrete implementation of Skyline queries in this setting.

Strengths

The algorithms are well described and supported by formal analysis of correctness and complexity. Experimental evaluations are comprehensive, covering both synthetic and real datasets.

The manuscript is very well organized, with logical flow, clear figures, and sufficient mathematical and algorithmic detail.

Data and methodology are transparent, and the inclusion of real-world web graph datasets enhances practical relevance.

Minor Points

A brief summary table comparing the proposed algorithms' theoretical complexities could further highlight their efficiency.

> We add Table 1 and Table 2 in the Algorithms section.

Minor language edits (e.g., article usage) could be handled during copyediting.

> We performed a complete review with respect to the language.
