# Supplementary material for: Computing the Skyline Query over a k2-tree compact data structure
Source: PLoS One. 2026 Jul 17;21(7):e0353358. doi: 10.1371/journal.pone.0353358 (PMC13379023; doi:10.1371/journal.pone.0353358)
Supplement: Peer review history

# Peer review: Decision Letter 0

**DOI:** 10.1371/journal.pone.0353358.r001

**Authors:** Anil Bhatia

13 Nov 2025

PONE-D-25-47139

Computing the Skyline Query over a k2-tree Compact Data Structure

PLOS ONE

Dear Dr. Caniupan,

Thank you for submitting your manuscript to PLOS ONE. After careful consideration, we feel that it has merit but does not fully meet PLOS ONE’s publication criteria as it currently stands. Therefore, we invite you to submit a revised version of the manuscript that addresses the points raised during the review process.

Please submit your revised manuscript by Dec 28 2025 11:59PM. If you will need more time than this to complete your revisions, please reply to this message or contact the journal office at plosone@plos.org. When you're ready to submit your revision, log on to https://www.editorialmanager.com/pone/ and select the 'Submissions Needing Revision' folder to locate your manuscript file.

Please include the following items when submitting your revised manuscript:

- A rebuttal letter that responds to each point raised by the academic editor and reviewer(s). You should upload this letter as a separate file labeled 'Response to Reviewers'.
- A marked-up copy of your manuscript that highlights changes made to the original version. You should upload this as a separate file labeled 'Revised Manuscript with Track Changes'.
- An unmarked version of your revised paper without tracked changes. You should upload this as a separate file labeled 'Manuscript'.

If you would like to make changes to your financial disclosure, please include your updated statement in your cover letter. Guidelines for resubmitting your figure files are available below the reviewer comments at the end of this letter.

If applicable, we recommend that you deposit your laboratory protocols in protocols.io to enhance the reproducibility of your results. Protocols.io assigns your protocol its own identifier (DOI) so that it can be cited independently in the future. For instructions see: https://journals.plos.org/plosone/s/submission-guidelines#loc-laboratory-protocols. Additionally, PLOS ONE offers an option for publishing peer-reviewed Lab Protocol articles, which describe protocols hosted on protocols.io. Read more information on sharing protocols at https://plos.org/protocols?utm_medium=editorial-email&utm_source=authorletters&utm_campaign=protocols.

We look forward to receiving your revised manuscript.

Kind regards,

Anil Bhatia, Ph.D

Academic Editor

PLOS ONE

**Journal Requirements:**

1. When submitting your revision, we need you to address these additional requirements.

Please ensure that your manuscript meets PLOS ONE's style requirements, including those for file naming. The PLOS ONE style templates can be found at

https://journals.plos.org/plosone/s/file?id=wjVg/PLOSOne_formatting_sample_main_body.pdf and

https://journals.plos.org/plosone/s/file?id=ba62/PLOSOne_formatting_sample_title_authors_affiliations.pdf

2. Please update your submission to use the PLOS LaTeX template. The template and more information on our requirements for LaTeX submissions can be found at http://journals.plos.org/plosone/s/latex.

3. Please note that PLOS One has specific guidelines on code sharing for submissions in which author-generated code underpins the findings in the manuscript. In these cases, we expect all author-generated code to be made available without restrictions upon publication of the work. Please review our guidelines at https://journals.plos.org/plosone/s/materials-and-software-sharing#loc-sharing-code and ensure that your code is shared in a way that follows best practice and facilitates reproducibility and reuse.

4. We note that the grant information you provided in the ‘Funding Information’ and ‘Financial Disclosure’ sections do not match.

When you resubmit, please ensure that you provide the correct grant numbers for the awards you received for your study in the ‘Funding Information’ section.

5. Thank you for stating the following financial disclosure:

ANID-FONDECYT # 1230647

Please state what role the funders took in the study.  If the funders had no role, please state: "The funders had no role in study design, data collection and analysis, decision to publish, or preparation of the manuscript."

If this statement is not correct you must amend it as needed.

Please include this amended Role of Funder statement in your cover letter; we will change the online submission form on your behalf.

6. Thank you for stating the following in the Acknowledgments Section of your manuscript:

This work is partially supported by ANID-FONDECYT # 1230647.

We note that you have provided funding information that is not currently declared in your Funding Statement. However, funding information should not appear in the Acknowledgments section or other areas of your manuscript. We will only publish funding information present in the Funding Statement section of the online submission form.

Please remove any funding-related text from the manuscript and let us know how you would like to update your Funding Statement. Currently, your Funding Statement reads as follows:

ANID-FONDECYT # 1230647

Please include your amended statements within your cover letter; we will change the online submission form on your behalf.

7. We note that your Data Availability Statement is currently as follows: All relevant data are within the manuscript and its Supporting Information files.

Please confirm at this time whether or not your submission contains all raw data required to replicate the results of your study. Authors must share the “minimal data set” for their submission. PLOS defines the minimal data set to consist of the data required to replicate all study findings reported in the article, as well as related metadata and methods (https://journals.plos.org/plosone/s/data-availability#loc-minimal-data-set-definition).

For example, authors should submit the following data:

- The values behind the means, standard deviations and other measures reported;

- The values used to build graphs;

- The points extracted from images for analysis.

Authors do not need to submit their entire data set if only a portion of the data was used in the reported study.

If your submission does not contain these data, please either upload them as Supporting Information files or deposit them to a stable, public repository and provide us with the relevant URLs, DOIs, or accession numbers. For a list of recommended repositories, please see https://journals.plos.org/plosone/s/recommended-repositories.

If there are ethical or legal restrictions on sharing a de-identified data set, please explain them in detail (e.g., data contain potentially sensitive information, data are owned by a third-party organization, etc.) and who has imposed them (e.g., an ethics committee). Please also provide contact information for a data access committee, ethics committee, or other institutional body to which data requests may be sent. If data are owned by a third party, please indicate how others may request data access.

8. Please amend either the abstract on the online submission form (via Edit Submission) or the abstract in the manuscript so that they are identical.

9. If the reviewer comments include a recommendation to cite specific previously published works, please review and evaluate these publications to determine whether they are relevant and should be cited. There is no requirement to cite these works unless the editor has indicated otherwise.

[Note: HTML markup is below. Please do not edit.]

Reviewers' comments:

Reviewer's Responses to Questions

**Comments to the Author**

1. Is the manuscript technically sound, and do the data support the conclusions?

The manuscript must describe a technically sound piece of scientific research with data that supports the conclusions. Experiments must have been conducted rigorously, with appropriate controls, replication, and sample sizes. The conclusions must be drawn appropriately based on the data presented.

Reviewer #1: Yes

Reviewer #2: Yes

Reviewer #3: Yes

**********

2. Has the statistical analysis been performed appropriately and rigorously?

Reviewer #1: Yes

Reviewer #2: Yes

Reviewer #3: Yes

**********

3. Have the authors made all data underlying the findings in their manuscript fully available?

The PLOS Data policy requires authors to make all data underlying the findings described in their manuscript fully available without restriction, with rare exception (please refer to the Data Availability Statement in the manuscript PDF file). The data should be provided as part of the manuscript or its supporting information, or deposited to a public repository. For example, in addition to summary statistics, the data points behind means, medians and variance measures should be available. If there are restrictions on publicly sharing data—e.g. participant privacy or use of data from a third party—those must be specified.

Reviewer #1: Yes

Reviewer #2: Yes

Reviewer #3: Yes

**********

4. Is the manuscript presented in an intelligible fashion and written in standard English?

PLOS ONE does not copyedit accepted manuscripts, so the language in submitted articles must be clear, correct, and unambiguous. Any typographical or grammatical errors should be corrected at revision, so please note any specific errors here.

Reviewer #1: Yes

Reviewer #2: Yes

Reviewer #3: Yes

**********

5. Review Comments to the Author

Please use the space provided to explain your answers to the questions above. You may also include additional comments for the author, including concerns about dual publication, research ethics, or publication ethics. (Please upload your review as an attachment if it exceeds 20,000 characters)

**Reviewer #1:**  The paper effectively outlines the problem and proposes novel algorithms to perform Skyline Queries directly on the k2-tree compact data structure. This innovation is clearly articulated, providing a strong foundation for the reader. However, more clarity could be provided on the specific computational benefits of the k2-tree in comparison to other compact data structures (e.g., quadtree or R-tree) in Skyline Query applications.

1. The claim of being the first implementation of Skyline Queries on compact structures needs stronger evidence and a comparative literature table.

2. Broader impact should be clarified through concrete application domains (e.g., large web-graph analytics, spatial decision support).

3. Quantify the real-world gain (e.g., % memory/time savings) to highlight significance.

4. However, Algorithms are clearly structured and theoretically justified with lemmas and complexity analyses. Provide explicit differentiation from prior BBS, R-tree, and bitmap-based Skyline methods.

5. I would suggest to include a summary table comparing structure type, need for decompression, time complexity, and memory overhead.

6. Add brief explanatory comments in pseudocode.

7. Provide a small, visual example (n = 8) showing how pruning and heap updates occur.

8. Present a table contrasting BBSk Constrained vs Skyline X (ordering criterion, pruning logic, expected complexity).

9. Summarize each lemma’s conclusion in plain language and include one table listing best, expected and worst-case complexities.

10. In Experimental Design- Add baseline comparison with a decompressed (plain) Skyline algorithm; Analyze scalability with respect to k and matrix size n.

11. Report standard deviations or error bars over the 10 × 10 runs.

12. Add numerical summaries below figures (e.g., “BBSK Manhattan achieved 45 % less memory than Skyline X”).

13. Discuss why Skyline X underperforms for uniform distributions yet matches performance for clustered ones.

14. Include a brief correctness check / validation against standard Skyline output to confirm identical results.

15. Add a short “Contributions” paragraph at the end of the Introduction summarizing: First Skyline computation on compact structure, Two Constrained Query variants, Enumerating Query algorithm with complexity proof, and Empirical performance gains.

16. Ensure consistent symbol usage (η, n, r, S) across pseudocode and text.

17. Check equation formatting (subscripts/superscripts) and figure captions.

18. The conclusion is concise, but further exploration into the practical implications of extending Skyline Queries to more than two dimensions could be discussed. Multi-dimensional Skyline Queries are a common challenge in many fields, and expanding the method to handle them efficiently would be a valuable contribution.

**Reviewer #2:**  The paper proposes algorithms to compute two variants of the Skyline Query—Constrained Skyline Query and Enumerating Skyline—directly on k²-tree compact representations of 2D point sets, without decompression. It builds on the Branch-and-Bound Skyline (BBS) approach, adapting it to k²-tree's recursive structure for efficient dominance checks and pruning. Theoretical analyses and experiments on synthetic and real datasets (Web graphs) demonstrate improved efficiency over non-indexed baselines.

Overall, the manuscript addresses a relevant gap in integrating multi-criteria queries with compact data structures, which is timely given the growth of large-scale spatial data. The k²-tree's application to Skyline Queries is innovative, and the direct computation on compressed data is a strength. However, the claimed "first study" overlooks the authors' own prior conference publication (cited as [7]), which reduces novelty for a journal extension. Methodological details are solid but could be enhanced for reproducibility (e.g., code availability). Clarity is good, though some sections (e.g., theoretical proofs) are dense. I recommend some revisions to address self-citation, expand novelty discussion, improve reproducibility, and refine analyses. Below, I detail major and minor concerns.

Major Concerns

1. Novelty and Relation to Prior Work: The abstract claims this is "the first study to implement Skyline Queries directly over compact data structures, specifically the k²-tree." However, this is an extension of the authors' own conference paper [7] (SCCC 2024), which already introduced these algorithms and experiments. While extensions are acceptable for PLOS ONE, the manuscript must explicitly discuss added contributions (e.g., deeper theoretical analyses, more proofs, expanded experiments) and cite [7] prominently in the abstract/introduction. Currently, [7] is mentioned briefly (p. 2), but the novelty claim is overstated—revise to emphasize journal-specific advancements. Also, discuss related work more broadly (e.g., skyline on other compacts like wavelet trees [web:3 from search], or distance joins on k²-tree ).

2. Reproducibility and Data Availability: PLOS ONE requires data/code sharing for verification. The manuscript lacks details on code implementation (e.g., C++ libraries for k²-tree, heap) or repository links. Synthetic data generation is described vaguely (e.g., uniform/clustered—provide parameters/algorithms). Real datasets are cited (LAW lab), but ensure direct access or deposition (e.g., in Figshare/Zenodo). Add a Data Availability Statement per PLOS guidelines, including scripts for experiments.

3. Theoretical Analysis Limitations: Proofs are correct but assume uniform/worst-case distributions without empirical validation (e.g., how often does the expected case O(k · log_k n + |S|) hold?). For Enumerating, the O(m · log n) counting cost (m = points) could dominate for dense datasets—discuss scalability thresholds. Clarify assumptions (e.g., k=2 in experiments; generalize to arbitrary k). Extend to higher dimensions (future work mentions this but suggest feasibility).

4. Experimental Scope: While comprehensive, add comparisons to index-based baselines (e.g., BBS on R-tree [2]) on decompressed data to quantify compression benefits. Real datasets are sparse (density <0.00034%), favoring k²-tree—test denser ones (e.g., geospatial points). Report standard deviations for averages (100 runs) to assess variability. Memory peaks are low (~MB), but profile heap vs. N array contributions in Compact Enumerating.

Minor Concerns

1. Clarity and Presentation: Some notations are inconsistent (e.g., "k2-tree" vs. "k²-tree"; use superscript). Proofs (e.g., Lemma 4) could use more steps for accessibility. Figures (e.g., 9-18) lack error bars; legends are clear but add units (e.g., µs/ms). English is good but polish awkward phrases (e.g., "the query remains relevant in the context of two-dimensional sets" → "remains applicable to 2D sets"). Add subheadings in sections (e.g., "Algorithm Description" vs. "Theoretical Analysis").

2. Related Work Gaps: Expand on skyline variants (e.g., dynamic skylines [9], probabilistic ) and k²-tree applications (e.g., graphs [6], set operations [22]). Discuss limitations of 2D focus (e.g., vs. higher-d k²-raster).

In summary, this is a sound contribution with practical value, but revisions are needed to clarify novelty as an extension, enhance reproducibility, and broaden comparisons. With these addressed, it would be suitable for publication. I look forward to the revised version.

**Reviewer #3:**  Summary

This manuscript presents a clear and rigorous study on implementing and evaluating Skyline Query variants directly over the k²-tree compact data structure. The authors successfully demonstrate that Constrained Skyline and Enumerating Skyline queries can be efficiently computed without decompressing data, achieving strong performance in both time and memory efficiency. The work bridges two active areas—compact data structures and multi-criteria query processing—and represents the first concrete implementation of Skyline queries in this setting.

Strengths

The algorithms are well described and supported by formal analysis of correctness and complexity. Experimental evaluations are comprehensive, covering both synthetic and real datasets.

The manuscript is very well organized, with logical flow, clear figures, and sufficient mathematical and algorithmic detail.

Data and methodology are transparent, and the inclusion of real-world web graph datasets enhances practical relevance.

Minor Points

A brief summary table comparing the proposed algorithms’ theoretical complexities could further highlight their efficiency.

Minor language edits (e.g., article usage) could be handled during copyediting.

Recommendation

This is a strong, well-written, and methodologically sound paper that makes a meaningful contribution to both data structure and database research. The experimental results convincingly support the conclusions, and the study meets PLOS ONE’s standards for technical soundness and clarity.

**********

6. PLOS authors have the option to publish the peer review history of their article (what does this mean?). If published, this will include your full peer review and any attached files.

If you choose “no”, your identity will remain anonymous but your review may still be made public.

**Do you want your identity to be public for this peer review?**  For information about this choice, including consent withdrawal, please see our Privacy Policy.

Reviewer #1: **Yes:**  Sonali Mishra

Reviewer #2: No

Reviewer #3: No

**********

[NOTE: If reviewer comments were submitted as an attachment file, they will be attached to this email and accessible via the submission site. Please log into your account, locate the manuscript record, and check for the action link "View Attachments". If this link does not appear, there are no attachment files.]

To ensure your figures meet our technical requirements, please review our figure guidelines: https://journals.plos.org/plosone/s/figures

You may also use PLOS’s free figure tool, NAAS, to help you prepare publication quality figures: https://journals.plos.org/plosone/s/figures#loc-tools-for-figure-preparation.

NAAS will assess whether your figures meet our technical requirements by comparing each figure against our figure specifications.

# Author comment: Author response to Decision Letter 1

**DOI:** 10.1371/journal.pone.0353358.r002

12 May 2026

We add a response to reviewers.

Attachment Submitted filename: answers to reviewers(v2).pdf

# Peer review: Decision Letter 1

**DOI:** 10.1371/journal.pone.0353358.r003

**Authors:** Anil Bhatia

24 Jun 2026

Computing the Skyline Query over a k2-tree Compact Data Structure

PONE-D-25-47139R1

Dear Dr. Caniupan,

We’re pleased to inform you that your manuscript has been judged scientifically suitable for publication and will be formally accepted for publication once it meets all outstanding technical requirements.

Within one week, you’ll receive an e-mail detailing the required amendments. When these have been addressed, you’ll receive a formal acceptance letter and your manuscript will be scheduled for publication.

An invoice will be generated when your article is formally accepted. Please note, if your institution has a publishing partnership with PLOS and your article meets the relevant criteria, all or part of your publication costs will be covered. Please make sure your user information is up-to-date by logging into Editorial Manager at Editorial Manager® and clicking the ‘Update My Information' link at the top of the page. For questions related to billing, please contact billing support.

If your institution or institutions have a press office, please notify them about your upcoming paper to help maximize its impact. If they’ll be preparing press materials, please inform our press team as soon as possible -- no later than 48 hours after receiving the formal acceptance. Your manuscript will remain under strict press embargo until 2 pm Eastern Time on the date of publication. For more information, please contact onepress@plos.org.

Kind regards,

Anil Bhatia, Ph.D

Academic Editor

PLOS One

Additional Editor Comments (optional):

Reviewers' comments:

Reviewer's Responses to Questions

**Comments to the Author**

1. If the authors have adequately addressed your comments raised in a previous round of review and you feel that this manuscript is now acceptable for publication, you may indicate that here to bypass the “Comments to the Author” section, enter your conflict of interest statement in the “Confidential to Editor” section, and submit your "Accept" recommendation.

Reviewer #1: All comments have been addressed

Reviewer #3: All comments have been addressed

**********

2. Is the manuscript technically sound, and do the data support the conclusions?

The manuscript must describe a technically sound piece of scientific research with data that supports the conclusions. Experiments must have been conducted rigorously, with appropriate controls, replication, and sample sizes. The conclusions must be drawn appropriately based on the data presented.

Reviewer #1: Yes

Reviewer #3: Yes

**********

3. Has the statistical analysis been performed appropriately and rigorously?

Reviewer #1: Yes

Reviewer #3: Yes

**********

4. Have the authors made all data underlying the findings in their manuscript fully available?

The PLOS Data policy requires authors to make all data underlying the findings described in their manuscript fully available without restriction, with rare exception (please refer to the Data Availability Statement in the manuscript PDF file). The data should be provided as part of the manuscript or its supporting information, or deposited to a public repository. For example, in addition to summary statistics, the data points behind means, medians and variance measures should be available. If there are restrictions on publicly sharing data—e.g. participant privacy or use of data from a third party—those must be specified.

Reviewer #1: Yes

Reviewer #3: Yes

**********

5. Is the manuscript presented in an intelligible fashion and written in standard English?

PLOS ONE does not copyedit accepted manuscripts, so the language in submitted articles must be clear, correct, and unambiguous. Any typographical or grammatical errors should be corrected at revision, so please note any specific errors here.

Reviewer #1: Yes

Reviewer #3: Yes

**********

6. Review Comments to the Author

Please use the space provided to explain your answers to the questions above. You may also include additional comments for the author, including concerns about dual publication, research ethics, or publication ethics. (Please upload your review as an attachment if it exceeds 20,000 characters)

Reviewer #1: All the comments have been properly incorporated in the present version of the manuscript. In my opinion, manuscript is ready for publication.

Reviewer #3: The authors addressed the major concerns raised during review. The authors have expanded the methodological and theoretical presentation, clarified the algorithmic descriptions with detailed examples and proofs, strengthened the experimental evaluation with both synthetic and real datasets, and added a meaningful comparison against R-tree–based approaches. The manuscript now presents a comprehensive and technically rigorous study of skyline query processing over compact k2-tree structures, with clear discussion of performance, scalability, and practical implications. The revisions improve both clarity and completeness, and the work makes a solid contribution to compact data structures and multidimensional query processing. I believe the manuscript is now ready for publication.

**********

7. PLOS authors have the option to publish the peer review history of their article (what does this mean?). If published, this will include your full peer review and any attached files.

If you choose “no”, your identity will remain anonymous but your review may still be made public.

**Do you want your identity to be public for this peer review?**  For information about this choice, including consent withdrawal, please see our Privacy Policy.

Reviewer #1: No

Reviewer #3: No

**********

# Editor report: Acceptance letter

**DOI:** 10.1371/journal.pone.0353358.r004

**Authors:** Anil Bhatia

PONE-D-25-47139R1

PLOS One

Dear Dr. Caniupan,

I'm pleased to inform you that your manuscript has been deemed suitable for publication in PLOS One. Congratulations! Your manuscript is now being handed over to our production team.

At this stage, our production department will prepare your paper for publication. This includes ensuring the following:

* All references, tables, and figures are properly cited

* All relevant supporting information is included in the manuscript submission,

* There are no issues that prevent the paper from being properly typeset

You will receive further instructions from the production team, including instructions on how to review your proof when it is ready. Please keep in mind that we are working through a large volume of accepted articles, so please give us a few days to review your paper and let you know the next and final steps.

Lastly, if your institution or institutions have a press office, please let them know about your upcoming paper now to help maximize its impact. If they'll be preparing press materials, please inform our press team within the next 48 hours. Your manuscript will remain under strict press embargo until 2 pm Eastern Time on the date of publication. For more information, please contact onepress@plos.org.

You will receive an invoice from PLOS for your publication fee after your manuscript has reached the completed accept phase. If you receive an email requesting payment before acceptance or for any other service, this may be a phishing scheme. Learn how to identify phishing emails and protect your accounts at https://explore.plos.org/phishing.

If we can help with anything else, please email us at customercare@plos.org.

Thank you for submitting your work to PLOS ONE and supporting open access.

Kind regards,

PLOS ONE Editorial Office Staff

on behalf of

Dr. Anil Bhatia

Academic Editor

PLOS One
